# Promoter RNA links transcriptional regulation of inflammatory pathway genes

**DOI:** 10.1093/nar/gkt777

**Published:** 2013-08-31

**Authors:** Masayuki Matsui, Yongjun Chu, Huiying Zhang, Keith T. Gagnon, Sarfraz Shaikh, Satya Kuchimanchi, Muthiah Manoharan, David R. Corey, Bethany A. Janowski

**Affiliations:** ^1^Department of Pharmacology and Biochemistry, UT Southwestern Medical Center, 6001 Forest Park Road, Dallas, TX 75390-9041, USA and ^2^Alnylam Pharmaceuticals, Cambridge, MA 02142, USA

## Abstract

Although many long non-coding RNAs (lncRNAs) have been discovered, their function and their association with RNAi factors in the nucleus have remained obscure. Here, we identify RNA transcripts that overlap the cyclooxygenase-2 (*COX-2*) promoter and contain two adjacent binding sites for an endogenous miRNA, miR-589. We find that miR-589 binds the promoter RNA and activates *COX-2* transcription. In addition to miR-589, fully complementary duplex RNAs that target the *COX-2* promoter transcript activate *COX-2* transcription. Activation by small RNA requires RNAi factors argonaute-2 (AGO2) and GW182, but does not require AGO2-mediated cleavage of the promoter RNA. Instead, the promoter RNA functions as a scaffold. Binding of AGO2 protein/small RNA complexes to the promoter RNA triggers gene activation. Gene looping allows interactions between the promoters of *COX-2* and phospholipase A2 (*PLA2G4A*), an adjacent pro-inflammatory pathway gene that produces arachidonic acid, the substrate for COX-2 protein. miR-589 and fully complementary small RNAs regulate both *COX-2* and *PLA2G4A* gene expression, revealing an unexpected connection between key steps of the eicosanoid signaling pathway. The work demonstrates the potential for RNA to coordinate locus-dependent assembly of related genes to form functional operons through *cis*-looping.

## INTRODUCTION

Many loci encode long non-coding RNAs (lncRNAs) that overlap the 5′ and 3′ termini of genes (1). Although the functional significance of many annotated nuclear lncRNAs remains under debate (2), recent reports have implicated them in the control of transcription (3–5). Understanding how lncRNAs may regulate gene transcription would provide insights into a mechanism that has the potential to supplement control by protein transcription factors. In addition, lncRNAs that regulate expression of key genes would be new targets for therapeutic intervention.

Well-studied examples of lncRNAs in mammalian cells include XIST and TSIX, RNAs that control X-chromosome inactivation (6), and HOTAIR, a lncRNA that contributes to silencing at the HOX locus (7). These RNAs appear to alter gene expression by functioning as scaffolds for histone modification complexes. Published models, however, cannot explain: (i) the basis for recognition at a specific gene, (ii) how small RNAs in concert with RNAi factors affect gene transcription, and (iii) how an lncRNA mediates sequence-specific control of gene expression.

LncRNAs functioning in *trans* induce effects at spatially distant loci. In contrast, action in *cis* is restricted to genes that are spatially close to where the lncRNA is transcribed. Localization of *cis*-acting lncRNAs to the chromatin near their sites of transcription may allow for the regulation of nearby genes. *Cis* interactions could, therefore, mediate a new layer of gene-specific regulation by complementary small RNAs and RNA-binding proteins to alter transcription, splicing, histone modification or local chromatin structure.

**Figure 1.**
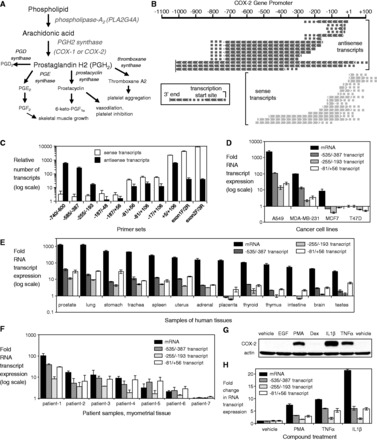
Transcription at the *COX-2* locus is bidirectional and coordinated. (**A**) Eicosanoid biosynthetic pathway. (**B**) Schematic of transcripts at the *COX-2* promoter identified by 5′ and 3′ RACE. (**C**) Identification and quantification of transcriptional domains at the *COX-2* promoter by strand-specific RT-qPCR. *n* = 3. (**D**) Expression of COX-2 mRNA or promoter RNA in cancer cell lines. *n* = 3. (**E**) Expression of COX-2 mRNA or promoter RNA in human tissues. (**F**) Expression of COX-2 mRNA or promoter RNA in myometrial tissue from patients. (**G**, **H**) Effect of adding pro-inflammatory agents on levels of COX-2 (**G**) protein, (**H**) mRNA and promoter RNA. Vehicle is solvent only control. Cells were treated with epidermal growth factor (10 ng/ml), PMA (50 ng/ml), IL1β (10 ng/ml) or TNFα (100 ng/ml) for 24 h. *n* = 3. Error bars are SD. Experiments use A549 cells unless otherwise noted. Westerns are representative of multiple experiments. See also Supplementary Figure S1.

To test whether *cis*-acting RNAs can alter gene transcription, we focused on the genomic locus encoding cyclooxygenase-2 (*COX-2*) and phospholipase A2 group IVA (*PLA2G4A*) (Figure 1A). COX-2 protein is a critical regulator of inflammation in normal physiology and disease. COX-2 catalyzes the conversion of arachidonic acid to intermediates that are further metabolized to prostaglandins and other eicosanoids (8). These eicosanoids mediate numerous biological processes including inflammation (9), development (10), reproduction (11), cellular immunity (12), cancer (13) and energy homeostasis (14). The *COX-2* promoter contains a TATA-box and numerous binding sites for transcription factors (15). Like many pro-inflammatory genes, transcription factors are recruited to these sites in different combinations depending on which signaling pathways are activated (16).

PLA2G4A is a cytosolic phospholipase that catalyzes the hydrolysis of membrane glycerophospholipids to release arachidonic acid, the substrate for the COX-2 enzyme (17). The gene encoding PLA2G4A is located on chromosome 1q25, adjacent to the *COX-2* locus. The *PLA2G4A* and *COX-2* promoters are organized head to head but are separated by 149 000 bases. The *PLA2G4A* promoter lacks SP1 sites and stretches of CpG islands that are characteristic of TATA-less promoters (18), suggesting that its expression may be influenced by other regulatory factors and pathways*.* Although the functions of COX-2 and PLA2G4A proteins are intimately linked, it is not known how their expression may be coordinated and how co-regulation of these pro-inflammatory genes would impact eicosanoid signaling.

The importance of COX-2 in normal physiology, disease and therapeutic development makes it a high-priority target for understanding gene regulation. Here, we address the role of RNA-directed interactions in regulating *COX-2* and *PLA2G4A* transcription. Unlike protein transcription factors that are general regulators of many genes, the selectivity inherent in RNA recognition permits sequence-selective control of gene targets. We discovered a network of RNAs overlapping the *COX-2* promoter leading us to investigate their impact on the expression of *COX-2* and *PLA2G4A*. Both genes are activated by endogenous miR-589 that targets sites within a COX-2 promoter RNA, demonstrating the potential for RNA to coordinate transcription of genes within the eicosanoid signaling pathway.

## MATERIALS AND METHODS

### mRNA-seq library generation and sequencing

A549 cells were first harvested, and pure nuclei were isolated. The nuclei were then dissolved in TRIzol, and total nuclear RNA was isolated subsequently following a standard TRIzol RNA preparation protocol (Sigma). Total nuclear RNA was treated with DNase I (Worthington) for 20 min at 37°C.

Illumina TruSeq mRNA-seq libraries were constructed according to manufacturer’s instructions (Illumina, Inc., San Diego, CA, USA) by starting with 1 µg of total RNA. To remove the most abundant duplex DNA species (representing more highly expressed transcripts), the mRNA-seq library was subjected to duplex-specific nuclease (DSN) treatment. In all, 280 ng of the TruSeq mRNA-seq library was treated with DSN according to manufacturer’s instructions (Evrogen/Axxora, LLC, San Diego, CA, USA) using a 2-h annealing period before DSN addition. Following DSN treatment, the library was subjected to an additional 14 cycles of PCR and post-PCR clean up using Agencourt AMPure XP beads (Beckman Coulter, Brea, CA, USA).

RNA-seq libraries were sequenced using the Illumina Hiseq 2000 as per manufacturer’s instructions for paired-end 2 × 100. In all, 108 million raw paired reads (100 nt) were obtained and then aligned to human genome hg19 using TopHat 1.4.1 (and Bowtie 0.12.8) by allowing up to two mismatches to the reference sequence in a paired-end mapping mode. Approximately, 71% of the raw reads were uniquely aligned (-g 1). The aligned reads were visualized in Integrative Genomics Viewer program.

### Small RNA-seq library generation and sequencing

A549 cells were harvested, and pure nuclei were isolated. The nuclei were then dissolved in TRIzol, and total nuclear RNA was isolated subsequently using Qiagen’s miRNeasy Mini Kit. Total nuclear RNA was treated with DNase I (Worthington) for 20 min at 37°C.

The small RNA-seq library was made following the standard Illumina small Truseq preparation protocol. RNA-seq libraries were sequenced using the Illumina Hiseq 2000 as per manufacturer’s instructions for single-end 1 × 50. Roughly, 50 million raw paired reads (averaged over two replicates) were obtained. Each raw read was trimmed by removing the adaptor sequence. The trimmed reads shorter than 15 nt were removed. The filtered trimmed reads were aligned to human genome hg18 using Bowtie 0.12.7 by allowing up to two mismatches to the reference sequence. Up to 10 different alignments per read was permitted. For reads that were aligned to multiple positions in the reference genome, the single aligned read with the fewest mismatches was selected using a Perl script. If there were still more than one aligned reads in this case, the original read would be disregarded. Approximately, 72% of the raw reads were successfully aligned. The aligned reads were again mapped to UCSC miRNA database and miRBase (mature miRNA) to search for possible miRNA hits.

### Rapid amplification of cDNA ends

Rapid amplification of cDNA ends (RACE) was performed using the GeneRacer Kit (Invitrogen). cDNA was prepared from total RNAs of A549 cells through reverse-transcription (RT) reaction using oligo dT or random primers. The 5′ or 3′ end of cDNA was amplified through two PCRs using Platinum Taq DNA polymerase (Invitrogen) and multiple primer sets (Supplementary Table S1). The thermal cycling condition for the first PCR was 94°C for 2 min, followed by 5 cycles of 94°C for 30 s and 72°C for 1 min, 5 cycles of 94°C for 30 s and 70°C for 1 min, and 25 cycles of 94°C for 30 s, 66°C for 30 s and 68°C for 1 min. The condition for the second nested PCR was 94°C for 2 min, followed by 25 cycles of 94°C for 30 s, 65°C for 30 s and 68°C for 1 min. PCR products were analyzed on 1% agarose gel (Supplementary Figure S1B). Major PCR products on gels were cloned and sequenced to determine transcription start sites or 3′ ends of transcripts (Supplementary Figure S1C).

### Quantitative PCR/strand-specific RT-qPCR

Total RNA from human cells and tissues were isolated using Tri-Reagent (Sigma). Two micrograms of total RNA were treated with DNase I to remove genomic DNA. Treated RNAs were reverse transcribed using the High Capacity cDNA Reverse transcription kit (Applied Biosystems). Random or strand-specific primer (exon 3 R or −740; 0.5 µM) was used in the RT reaction. Quantitative PCR (qPCR) was performed using iTaq SYBR Green Supermix (Biorad) with 50 ng of cDNA as template. Data were normalized relative to measured GAPDH levels. Primers used in qPCR are listed in Supplementary Table S1.

### Cellular delivery of duplex RNAs, single-stranded gapmers and locked nucleic acid miR inhibitors

Lipofectamine RNAiMAX (Invitrogen) was used in reverse transfection experiments (i.e. lipid/RNA mixtures added to a culture well before cells) to deliver duplex RNAs, single-stranded gapmers or locked nucleic acid (LNA) anti-miRs into A549 cells as described (19). Cells transfected with duplex RNAs were harvested 3 days after transfection for qPCR and 4–5 days after transfection for western blot. In double transfection experiments with duplex RNAs, the second transfection was performed 2 days after the first transfection, and cells were harvested on day 5. Sequences of duplex RNAs and gapmers are listed in Supplementary Tables S2 and S3. Anti-miRs complementary to miR-589 and -452 were obtained from Exiqon. Transfection of single-stranded gapmers and LNA anti-miRs required a double transfection. Reverse transfections were performed in 6-well plates using 125k A549 cells/well. Three days after the first transfection, cells were counted and reverse transfected a second time. Cells were harvested 3 days later for western blot and qPCR. Concentrations for each experiment are provided in the figure legends. Transfection of T47D required 200k cells/well.

### Western analysis

Cells were harvested with trypsin-EDTA solution (Invitrogen) and lysed. The protein concentration in each sample was quantified by BCA assay (Thermo Scientific). SDS–PAGE was performed using 7.5% Tris–HCl, 7.5% TGX or 4–20% TGX gels (Biorad). Gels were run at 100 V for 70 min. After gel electrophoresis, proteins were transferred to nitrocellulose membrane (Hybond-C Extra; GE Healthcare Life Sciences) at 100 V for 90 min. Membranes were incubated with specific primary antibodies at the following dilution ratios: anti-COX-2 antibody (1:2500; 160 112; Cayman Chemical), anti-cPLA2 (PLA2G4A) (1:2000; 5479S; Cell Signaling), anti-β-Actin (1:20 000; A5441; Sigma), anti-AGO2 (1:1000; ab57 113; Abcam) and anti-GW182 (1:4000; A302–329A; Bethyl Labs). Horseradish peroxidase-conjugated anti-mouse (715-035-150) or anti-rabbit (711-035-152) secondary antibody (1:4000–1:20 000; Jackson Immunolabs) was used for visualizing proteins using SuperSignal West Pico Chemiluminescent Substrate (Thermo Scientific).

### Melting temperature (*T*_m_) determination

Annealed duplex RNAs were diluted to 1.5 μM in 0.1 M NaH_2_PO_4_/Na_2_HPO_4_ buffer (pH 7.4) (final volume 400 μl) and transferred to quartz cuvettes. The solutions were topped with mineral oil (300 μl) and heated from 15 to 95°C at 1°C/min while monitoring changes in A260 using a Cary 100 Bio spectrophotometer. Samples were then re-annealed in the spectrophotometer, and the experiment was repeated several times. *T*_m_ values were calculated as the first derivatives of the plot of A260 versus temperature, and the results from three to four experiments were averaged.

### Treatment of untransfected/transfected cells with physiologic agents

Two days after plating, cells were treated with epidermal growth factor (EGF; 10 ng/ml), phorbol 12-myristate 13-acetate (PMA; 50 ng/ml), interleukin1-beta (IL1β; 10 ng/ml), tumor necrosis factor-alpha (TNFα; 100 ng/ml), dexamethasone (Dex; 1 µM) or vehicle (water or DMSO) in media containing 2% charcoal-stripped FBS (Atlanta Biologicals) for 24 h then harvested for qPCR or western blot. For combination treatment with RNA12 (25 nM) or RNA12*nc* (25 nM), compounds were added to cells 2 days after transfection in media containing 2% charcoal-stripped FBS and harvested 24 h later for RNA. qPCR was used to evaluate levels of mRNA and lncRNA.

### Chromatin immunoprecipitation/RNA immunoprecipitation

Chromatin immunoprecipitation (ChIP) and RNA immunoprecipitation (RIP) assays were performed as previously described (20,21). A549 cells (2 000 000 cells in 15-cm dish) were reverse-transfected with RNA12, RNA12*nc* or mismatch oligomer (25 nM) on Day 0. Untreated cells were also prepared on Day 0. The cells were treated with either vehicle or 10 ng/ml IL1β for 1 h on day 3. For most ChIP and RIP cells were crosslinked with 1% formaldehyde for 10 min, whereas no crosslink protocol (20) was used for AGO2-RIP, and 0.1% formaldehyde was used for GW182-RIP. Cells were recovered by scraping, and nuclei were isolated using hypotonic lysis (HL) buffer [4 ml; 10 mM Tris–HCl (pH 7.5), 10 mM NaCl, 3 mM MgCl_2_, 0.5% (v/v) NP-40]. Nuclei were lysed in lysis buffer [1 ml; 1% SDS, 10 mM EDTA, 50 mM Tris–HCl (pH 8.1), 1xRoche protease inhibitors cocktail, RNasin Plus RNase inhibitor (Promega)] and sonicated (2 pulse, 20% power setting, 20 s).

Nuclear lysate (40–250 µl) was incubated overnight with specific antibodies (2–5 µg) in immunoprecipitation buffer. After antibody-protein-DNA/RNA complex was recovered with 40–50 µl of protein G plus/protein A agarose beads (Calbiochem), the beads were washed with buffers. The complex was eluted twice with 250 µl of elution buffer (1% SDS, 0.1 M NaHCO_3_). Cross-linking was reversed by adding NaCl to a final concentration of 200 mM and heating at 65°C for at least 2 h. Protein was digested by incubating with proteinase K (20 µg; invitrogen) at 42°C for 50 min, followed by phenol-chloroform extraction and ethanol precipitation. For ChIP, samples were dissolved in 200 µl of nuclease free water and analyzed by qPCR. For RIP, samples were dissolved in 17.6 µl of nuclease free water and treated with DNase I at 25°C for 20 min. RT reactions (20 µl) were performed for input and +RT samples, then the samples were analyzed by qPCR. PCR products were analyzed by 2.5% agarose gel electrophoresis, and gels were stained with ethidium bromide.

Antibodies used in ChIP/RIP were as follows: anti-RNAP2 (05-623, Millipore), anti-H3K4me3 (ab8580, Abcam), anti-H3K27me3 (07-449, Millipore), anti-H4Ac (06-598, Millipore), anti-NFκB p65 (17-10 060, Millipore), anti-NFκB p50 (06-886, Millipore), anti-CREB1 (17-600, Millipore), anti-AGO2 (015-22 031, Wako), anti-WDR5 (A302-429A, Bethyl Labs) and anti-GW182 (A302-329A, Bethyl Labs). Normal mouse immunoglobulin G (IgG) (12-371, Millipore) or normal rabbit IgG (12-370, Millipore) was used as a negative control. The sequences of qPCR primers used in ChIP or RIP are listed in Supplementary Table S1.

### Cell fractionation and extract preparation

A549 cells were transfected with 25 nM RNA12, RNA12*nc* or mismatched control duplex (MM) using Lipofectamine RNAiMAX and harvested 24 h later. Cells were fractionated into cytoplasmic and nuclear extracts. Cells were first resuspended in HL buffer [10 mM Tris (pH 7.4), 10 mM NaCl, 3 mM MgCl_2_, 0.3% NP-40, 1% protease inhibitor cocktail] then incubated on ice for 15 min and the soluble fraction kept as cytoplasmic extract. To the cytoplasmic extract, KCl and glycerol were added to a final of 140 mM and 10%, respectively. Nuclei in the pellet were washed 3× with HL buffer then resuspended in either nuclear lysis buffer [20 mM Tris (pH 7.4), 0.15 M KCl, 3 mM MgCl_2_, 0.5% NP-40, 10% glycerol, 1% protease inhibitor cocktail]. Nuclei resuspended in nuclear lysis buffer were sonicated on ice at 20% power 3× for 20 s. The soluble fraction was kept as nuclear extract. All extracts were stored at −80°C for later use.

Samples from whole cell, cytoplasmic and nuclear fractions were analyzed by western blot to show cellular localizations of AGO2 and GW182. Western analysis was performed using anti-AGO2 (1:1000; ab57 113; abcam), anti-GW182 (1:4000; A302-329A; Bethyl Labs), anti-tubulin (1:6000; T5201; Sigma), anti-calreticulin (1:1000; 2891S; Cell Signaling) or anti-histone H3 (1:10 000; 2650S; Cell Signaling) primary antibody and horseradish peroxidase-conjugated anti-mouse IgG (715-035-150) or anti-rabbit IgG (711-035-152) secondary antibody (1:5000–1:10 000; Jackson Immunolabs) as described earlier in the text.

### Co-immunoprecipitations

Co-immunoprecipitation experiments were performed by mixing 30 µl of Protein G Plus/Protein A resin, 1.5 µg of antibody (anti-AGO2, ab57113, Abcam; anti-GW182, Bethyl Laboratories) and nuclear extract (0.5–1 mg of total protein) and incubating at 4°C for 4 h. Resin was washed 4x with IP wash buffer [20 mM Tris (pH 7.5), 0.4 M NaCl, 2 mM MgCl_2_, 0.05% NP-40, 0.025% SDS] and co-purified proteins eluted by boiling resin in 25 µl 1× SDS loading buffer. Elution was resolved by SDS–PAGE, and proteins detected by western blot with anti-AGO2 or anti-GW182 antibodies.

### AGO2 cleavage assay

AGO2 protein was immunoprecipitated from each extract using 1.5 µg of anti-AGO2 antibody (Abcam, ab57113) and Protein G Plus/Protein A agarose. Target RNA substrate RNA12*Sub* was 5′ radiolabeled and gel-purified. Labeled RNA12*Sub* (100 000 cpms, ∼0.2 pmols) was added to the resin in 1× RNAi buffer [50 mM Tris (pH 7.4), 2 mM MgCl_2_, 0.5 mM DTT, 100 mM KCl, 50 mM NaCl, 1 mM EDTA] supplemented with 0.5 µg/µl yeast tRNA and 2.5 mM ribonucleoside triphosphates. Cleavage reactions were incubated at 30°C for 1.5 h, and RNA was collected by phenol extraction. Extracted RNA was precipitated with 9 volumes of 2% LiClO_4_ in acetone, washed with acetone, resuspended in 90% formamide, 1× TBE buffer and resolved on a 14% denaturing polyacrylamide (7 M urea, 1× TBE). Gels were dried and exposed to phosphorimager screens overnight to visualize radioactivity. RNA12*Sub* consists of a single siRNA target site flanked by firefly luciferase gene sequence and capped with two deoxynucleosides on each end. RNA12*Sub* sequence (siRNA target site capitalized): gaacaauugauuuuacagacGACAAUUGGUCGCUAACCGucacguacgcggaauacutc.

### Chromosome conformation capture (3C) assay

A549 cells (2 million cells per 15-cm dish) were reverse-transfected with RNA12 or control mismatch duplex. Samples were treated with 2% formaldehyde for 10 min for cross-linking. No cross-link samples were also prepared as negative controls. Nuclei were harvested using HL buffer [10 mM Tris–HCl (pH 7.5), 10 mM NaCl, 3 mM MgCl_2_, 0.5% (v/v) NP-40]. Three million nuclei were resuspended in 500 µl of 1.2×restriction enzyme buffer containing 0.1% (w/v) SDS and incubated at 65°C for 8 min to loosen chromatin. To sequester SDS, Triton X-100 was added up to 1% (v/v). Samples were treated with 250 U of *Dpn*II (NEB) at 37°C overnight. The next day, SDS was added to 1.6% (v/v) and heated at 65°C for 20 min to inactivate the restriction enzyme followed by adding 1% (v/v) Triton X-100. For ligation, samples were incubated with 4000 cohesive end units of T4 DNA ligase (NEB) at 16°C for 5 h. Samples were then treated with 200 mM NaCl and 300 µg of proteinase K at 65°C overnight for reverse cross-linking. After treating samples with 40 ng/µl RNase A (300 µg) at 37°C for 40 min, DNA fragments were purified by phenol-chloroform extraction and ethanol precipitation. The 3C products were further purified using Wizard SV Gel and PCR clean-up system (Promega). Purified 3C samples were diluted with nuclease-free water and used for qPCR. iTaq SYBR Green (Biorad) was used for constant primer C1 or C3 and PrimeTime qPCR Assay (IDT) was used for constant primer C2 in 3C-qPCR. Primers and a probe used in 3C experiments are listed in Supplementary Table S1. Digestion efficiency evaluated by qPCR amplifying across the restriction sites was ∼90%. All 3C-qPCR products were confirmed by sequencing.

## RESULTS

### Sense and antisense promoter RNAs are transcribed across the COX-2 locus

We sequenced polyA-selected RNA from the nuclei of A549 non-small cell lung cancer cells to identify candidate genes that express long RNAs overlapping their promoters. We obtained 76 million paired-end (2 × 100) reads that were uniquely aligned to human genome-19. We observed reads spanning the *COX-2* promoter (Supplementary Figure S1A) and then investigated transcription at the *COX-2* locus in more detail.

We defined the origin and orientation of the transcripts using 5′ and 3′ RACE (Supplementary Figure S1B) and observed divergent sense and antisense domains of transcription at the *COX-2* promoter (Figure 1B, Supplementary Figure S1C). The *COX-2* promoter is controlled by a TATA box and mRNA transcription initiates almost entirely at the annotated +1 site. To quantify RNA levels, we used strand-specific RT-qPCR and found that sense transcription was prevalent near the transcription start site, and antisense transcription was predominant upstream (Figure 1C).

Both droplet digital PCR and qPCR showed that the sense transcripts upstream of the transcription start site were present in approximately two copies per cell (22). Transcript distribution was similar in cell nuclei and cytoplasm. It is possible that the sense transcripts encode variants that can be translated into COX-2 protein. Protein produced by upstream variant transcripts, however, would be low relative to protein produced from mRNA that initiates at the +1 transcription start site. These data reveal an RNA network overlapping key regulatory regions in the *COX-2* promoter.

### Coding and non-coding transcription at the COX-2 locus is coordinated

The presence of an RNA network at the *COX-2* promoter suggested a possible role for promoter RNA to regulate *COX-2* gene expression. To investigate this possibility, we measured promoter RNA and mRNA expression in A549 lung cancer cells and in the breast cancer cell lines, MDA-MB-231, MCF7 and T47D. These cell lines were chosen because their basal levels of *COX-2* expression differ, ranging from moderate to very low. We used primer sets to detect RNA levels within the upstream domain of antisense transcription (primer set −535/−387), a middle region where less transcription occurs (primer set −255/−193) and the downstream domain of transcription (primer set −81/+56).

Expression of COX-2 mRNA and promoter RNA varied similarly to one another in the cell lines and human tissues (Figure 1D and E). *COX-2* expression in myometrial tissue is elevated just before parturition and provides a classic example of acute *COX-2* upregulation in response to physiologic stress (23). In patient samples, increased expression of both sense and antisense RNAs was associated with increased expression of COX-2 mRNA (Figure 1F).

The *COX-2* promoter is responsive to endogenous signals including pro-inflammatory cytokines, growth factors and hormones. Treatment of A549 lung cancer cells with the *COX-2* gene activators, PMA, IL1β and TNFα increased levels of COX-2 protein (Figure 1G), mRNA and promoter RNA (Figure 1H). Combined, these results from stimuli-treated cells, cancer cells, patient samples and human tissues all support the conclusion that expression of COX-2 mRNA and promoter RNA domains vary in a coordinated and potentially co-regulated fashion.

### Endogenous nuclear miR-589 controls expression of *COX-2*

It is not clear how nuclear non-coding RNAs associated with promoters might alter gene expression. We hypothesized that nuclear miRNAs could recognize promoter RNA and affect transcription of *COX-2*. To address this hypothesis, we first performed deep sequencing of nuclear RNA from A549 cells to reveal the identity of highly expressed miRNAs. We then performed computational searches to identify miRNAs that were expressed in A549 cells and possessed seed sequence complementarity to RNA at the *COX-2* promoter.

**Figure 2.**
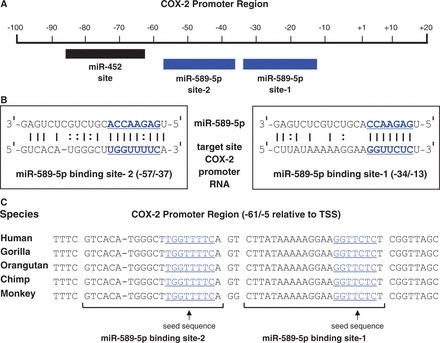
The COX-2 promoter RNA contains two binding sites for miR-589-5p. (**A**) Binding sites for miR-452 and miR-589-5p relative to the *COX-2* promoter. (**B**) The two target sites for miR-589-5p within the sense COX-2 promoter RNA. Bold and underlined: seed sequences. (**C**) Sequence conservation of binding sites for miR-589-5p in primates. Underlined: seed sequences.

**Figure 3.**
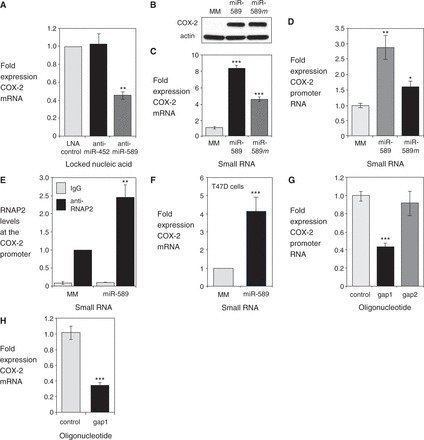
miR-589 activates transcription of the *COX-2* gene. (**A**) qPCR of COX-2 mRNA after transfection of anti-miRs (42 nM) that target miR-589 or miR-452. LNA control is not complementary to either sequence. *n* = 3. (**B**, **C**) Western or qPCR detection of COX-2 protein or mRNA after treatment with mature miR-589 (miR-589, 42 nM) or miR-589 mimic (miR-589 *m,* 42 nM). *n* = 3. (**D**) qPCR data showing effect of mature miR-589 (miR-589, 42 nM) or mimic (miR-589 *m*, 42 nM) on expression of COX-2 promoter RNA (−81/+56). *n* = 2. (**E**) ChIP demonstrating recruitment of RNAP2 to the *COX-2* promoter on addition of mature miR-589 (miR-589, 42 nM). *n* = 3. (**F**) qPCR of COX-2 mRNA on addition of mature miR-589 (miR-589, 42 nM) in T47D cells. *n* = 2. (**G**) qPCR showing reduction in sense promoter RNA (−81/+56) levels after transfection of a complementary gapmer oligonucleotide (gap1, 42 nM). Control is a single-stranded gapmer oligonucleotide with no complementarity to the COX-2 promoter RNA. *n* = 3. (**H**) qPCR showing reduction in COX-2 mRNA levels after transfection of a complementary gapmer oligonucleotide (gap1, 42 nM). Control is a single-stranded gapmer oligonucleotide with no complementarity to the COX-2 promoter RNA. *n* = 2. MM = mismatched RNA control. Error bars are SD. *P < 0.05, **P < 0.01, and ***P < 0.001 (*t*-test) relative to control.

miR-589 was a top candidate because one strand of miR-589, miR-589-5p, possessed seed sequence complementarity to two adjacent sequences within the sense promoter RNA (Figure 2A and B). Both putative target sites are perfectly conserved in primates (Figure 2C). We obtained anti-miRs, short single-stranded LNA oligonucleotides, to investigate the endogenous function of miR-589 on *COX-2* gene expression. Treatment with the anti-miR complementary to miR-589-5p resulted in reduced basal expression of *COX-2* (Figure 3A). This result suggested that miR-589-5p may be an endogenous activator of *COX-2*. By contrast, an anti-miR complementary to miR-452 (an miRNA with just one putative binding site within the *COX-2* promoter, Figure 2A) had no effect on basal *COX-2* expression.

We then examined how introducing miR-589 into A549 cells would affect *COX-2* expression. When miR-589 was added as either mature miRNA with two partially complementary strands or as a miRNA mimic with two fully complementary strands, we observed increased levels of COX-2 protein (Figure 3B) and mRNA (Figure 3C). This increased expression of *COX-2* further characterizes miR-589 as an endogenous activating factor in the nucleus.

Although fully complementary siRNAs cause target transcript levels to decrease, miR-589 has central mismatches relative to its target sites within the COX-2 promoter RNA, which should prevent transcript cleavage by RNAi factors (24). As predicted, treatment with miR-589 did not decrease levels of the promoter RNA. Instead, promoter RNA levels increased by 1.5–3-fold (Figure 3D). The increase in promoter transcript levels may reflect a general increase in transcription at the *COX-2* locus that is not limited to increased production of mRNA. Recruitment of RNA polymerase 2 (RNAP2) to the *COX-2* promoter also increased (Figure 3E) in nuclei harvested from cells treated with miR-589, consistent with miR-589 activating *COX-2* transcription.

To examine the action of miR-589 in a different cellular context, we introduced miR-589 into T47D breast cancer cells. Unlike A549 cells, expression of miR-589 is undetectable, and *COX-2* expression is substantially lower. We found that addition of miR-589 increased COX-2 mRNA levels ∼4-fold (Figure 3F). These data show that RNA-mediated activation of *COX-2* expression can occur over a wide range of basal expression levels.

We next sought to determine whether promoter RNA levels were directly linked to *COX-2* expression by reducing expression of the promoter RNA using a gapmer oligonucleotide. Gapmers are antisense oligonucleotides with chemically modified flanking regions to increase affinity and a central DNA region to recruit RNase H for efficient cleavage of DNA-RNA hybrids (25). Gapmers are single-stranded and can selectively reduce the expression of either sense or antisense promoter RNAs.

We identified a gapmer oligonucleotide (gap1) that reduced levels of the sense promoter RNA, and a gapmer complementary to the promoter antisense strand (gap2) that did not (Figure 3G). When gap1 reduced levels of the sense promoter RNA, expression of COX-2 mRNA was also reduced (Figure 3H). Alterations in *COX-2* expression following treatment with anti-miR, miRNA mimic or gapmer are all consistent with the sense promoter RNA acting as a docking site for miR-589. Both promoter RNA and miR-589 are required for RNA-mediated activation of *COX-2* expression.

### Transcriptional activation of the *COX-2* gene by miR-589 involves the RNAi factors, AGO2 and GW182

Since small RNAs require RNAi proteins to function, we reasoned that the RNAi factor argonaute-2 (AGO2) protein might be involved in RNA-mediated activation of *COX-2*. It is well known that AGO2 promotes binding of small RNAs to RNA transcripts during cytoplasmic RNAi. AGO2 is also found in cell nuclei of yeast, plants and animals (26). RNAi has been reported to function in mammalian nuclei (27) where AGO2 can facilitate RNA-mediated modulation of transcription (20,21) or splicing (28).

**Figure 4.**
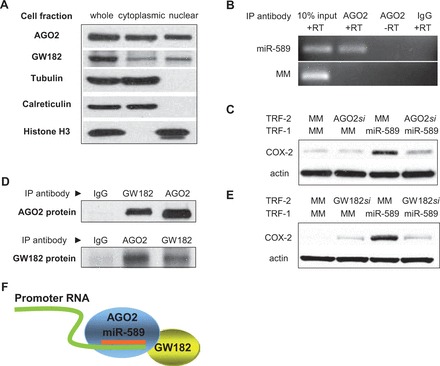
Activation of the *COX-2* gene by miR-589 requires AGO2 and GW182. (**A**) Identification of GW182 and AGO2 proteins in mammalian cell nuclei. Tubulin is a cytoplasmic marker, calreticulin is an ER marker, and histone H3 is a nuclear marker. (**B**) RIP showing recruitment of AGO2 protein to promoter RNA (−81/+56) by mature miR-589 (miR-589, 42 nM). (**C**) Western showing effect of reducing cellular AGO2 levels on activation of *COX-2* expression by mature miR-589 (miR-589, 30 nM). AGO2*si* is a pool of duplex RNAs (12 nM) complementary to AGO2 mRNA. (**D**) Co-immunoprecipitation of AGO2 protein (top) or GW182 protein (bottom) from mammalian nuclei using anti-AGO2 or anti-GW182 antibodies. (**E**) Western showing effect of reducing cellular GW182 levels on activation of *COX-2* expression by mature miR-589 (miR-589, 30 nM). GW182*si* is a pool of duplex RNAs (12 nM) that reduces expression of all three cellular GW182 paralogs. (**F**) Scheme showing association of GW182, AGO2 and promoter RNA. All experiments were performed in A549 cells. MM, mismatched RNA control; TRF, transfection; RT. reverse transcription. Westerns are representative of multiple experiments.

Using stringent cellular fractionation protocols followed by western blot analysis, we found that both AGO2 and a second RNAi factor, GW182 (29), are present in the nuclei of A549 cells (Figure 4A). We performed RIP from nuclear extracts using an anti-AGO2 antibody to determine whether AGO2 associates with promoter RNA at the *COX-2* promoter. RIP revealed the recruitment of AGO2 to the COX-2 promoter RNA was dependent on the addition of miR-589 (Figure 4B).

To further investigate a potential role for AGO2 in transcriptional activation by small RNA, we used anti-AGO2 siRNAs to deplete AGO2 levels inside cells and examined how reduction of AGO2 expression would affect gene activation. We observed that RNA-mediated gene activation by miR-589 was blocked when AGO2 levels were reduced (Figure 4C). Similar experiments using an RNA with seed sequence mismatches did not block gene activation or cause AGO2 recruitment. These two complementary experimental approaches, RIP and AGO2 depletion, support the conclusion that AGO2 is required for the sequence-selective recognition of miR-589 to the promoter RNA and support a role for nuclear RNAi factors in endogenous transcriptional regulation.

GW182 (TNRC6A) is an important factor in the mechanism of translational silencing by miRNAs (29). GW182 interacts directly with AGO2 through protein:protein interactions (30–32). Co-immunoprecipitations with anti-AGO2 or anti-GW182 antibody revealed that AGO2 and GW182 form a complex in stringently purified cell nuclei (Figure 4D). To determine whether activation of *COX-2* expression requires GW182, we used an siRNA pool capable of reducing expression of all three GW182 paralogs (Supplementary Table S2). Reducing GW182 expression reversed miR-589-mediated activation of *COX-2* expression relative to A549 cells that had been treated with a control RNA that left GW182 levels unchanged (Figure 4E).

These results indicate that the RNAi factors AGO2 and GW182 associate in the nucleus (Figure 4D and F) and are required for transcriptional activation of the *COX-2* gene by miR-589 (Figure 4C and E). Taken together, the results suggest that AGO2 forms a complex with GW182 and miR-589 that is guided to the promoter RNA. Binding specificity is achieved through Watson–Crick base-pairing of miR-589 to the promoter RNA (Figure 4F).

### Discovery of more potent activating small RNAs

Activation of *COX-2* gene expression by miR-589 suggested that it would be possible to discover related small RNAs that were more potent modulators of gene expression. Selective and robust RNA activators would be transformative as: (i) tools to investigate the mechanism of RNA-mediated gene activation (higher levels of activation increase signal to noise and facilitate the diverse experiments); (ii) facilitators for gain of function studies in the same manner that RNAi knock-down enables loss of function experiments; and (iii) new options in the development of therapeutics where upregulation of a specific gene is desired. Duplex RNAs that are fully complementary to gene promoters have been reported to activate several genes, suggesting that modulation of transcription by small RNAs may be a general regulatory mechanism (5,20,21,33–38).

**Figure 5.**
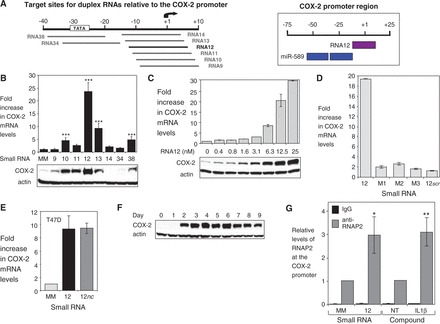
A fully complementary RNA also activates *COX-2* expression. (**A**) Target sites for duplex RNAs relative to the *COX-2* promoter (left). Target sites for miR-589 and RNA12 relative to the *COX-2* promoter (right). (**B**) qPCR and western data showing the effect of duplex RNAs (25 nM) on levels of COX-2 mRNA (upper, *n* = 3) and protein (lower). (**C**) qPCR and western data showing the effect of increasing concentrations of RNA12 (0–25 nM) on COX-2 mRNA (upper) and protein (lower) expression. (**D**) qPCR detection of COX-2 mRNA after transfection of RNA12 or duplex RNAs that contain mismatched (M1, M2, M3) or scrambled bases (12*scr*) (25 nM). *n* = 3. (**E**) qPCR data showing activation of *COX-2* expression by RNA12 or RNA12*nc* (25 nM) in T47D cells. MM, mismatched RNA control. (**F**) Western showing time-course of COX-2 protein expression after transfection of RNA12 (25 nM). (**G**) ChIP showing recruitment of RNAP2 at the *COX-2* promoter after addition of RNA12 (25 nM) or IL1β (10 ng/ml). *n* = 4. Error bars are SD. *P < 0.05, **P < 0.01 and ***P < 0.001 (*t*-test) relative to MM or no treatment (NT).

We designed small RNAs complementary to sequences adjacent to or overlapping the target sites of miR-589 within the COX-2 promoter RNA (Figure 5A). In contrast to miR-589, these duplex RNAs had a single target site and were fully complementary. We found that several of these small RNAs activated *COX-2* expression (Figure 5B).

One of these RNAs, RNA12, achieved 25-fold activation, significantly more than the 8-fold enhancement achieved by miR-589. Activation was potent with half maximal activation achieved at <10 nM(Figure 5C). The seed sequence necessary for efficient recognition by small RNAs during RNAi occupies bases 2–8 within the guide strand of a duplex RNA (39). The introduction of one or more mismatched bases into the seed sequence of RNA12 dramatically reduced activity (Figure 5D), consistent with the promoter RNA being the molecular target. Like miR-589, RNA12 activated *COX-2* expression in T47D cells (Figure 5E). Combined treatment of RNA12 and miR-589 did not result in additive activation of *COX-2* expression (Supplementary Figure S2A). Activation was transient, decreasing after 7 days (Figure 5F). Although transient, the activation was also persistent, as it remained detectable after 9 days.

Although RNA12 was the strongest activator of *COX-2* expression, it was not the only RNA activator discovered in our screen. Several similar RNAs also activate expression by as much as 5–10-fold (Figure 5B, RNA10, RNA13, RNA38). One of these RNAs, RNA38, possesses a seed sequence that overlaps with miR-589. Other RNAs were less active, consistent with previous observations that closely related nucleic acid sequences can vary in activity (40). This variation may be due to differences in the accessibility of the target RNA, association or usage by RNAi factors or the ability to form productive interactions at the promoter. We have previously observed that, when introduced into cells first, inactive duplex RNAs that are fully complementary to a promoter transcript blocked gene upregulation of progesterone receptor by an activating RNA with an overlapping target site (34). This result shows that the exact location for recognition matters during activation.

The large dynamic range in gene activation by RNA12 made it an ideal small RNA for evaluating mechanism. RNA12 treatment increased COX-2 pre-mRNA levels, a result consistent with increased gene transcription (Supplementary Figure S2B). ChIP from purified nuclei revealed that activation by RNA12 induced recruitment of RNAP2 (Figure 5G), CREB1 and NFκB (p65, p50) (Supplementary Figure S3) to the *COX-2* promoter. Recruitment of NFκB and CREB1 on activation of the *COX-2* gene is also consistent with induction of transcription by RNA12 (16). Taken together, these observations provide support for the existence of an RNA-mediated mechanism that can activate gene transcription.

When RNA strands are introduced into cells, they can provoke an interferon response and that might induce *COX-2* expression. To address this possibility, we assayed the activity of an RNA12 variant (RNA12*m m* = modified). Like RNA12, RNA12*m* is fully complementary to its target but differs by introduction of chemical modifications known to minimize the interferon response (41). RNA12*m* activated *COX-2* expression by ∼10-fold (Supplementary Figure S2C), demonstrating that RNA designs compatible with *in vivo* development are effective activators of gene expression. Levels of interferon responsive genes after treatment with RNA12 or RNA12*m* did not significantly increase (Supplementary Figure S4A and B). These data, together with stringent discrimination against activation by duplexes with seed sequence mismatches, strongly support the hypothesis that small RNAs targeted to the *COX-2* transcription start site are ‘on-target’ activators of *COX-2* gene expression.

### Small RNAs mediate a distinct pathway for *COX-2* gene activation

*COX-2* expression can be activated by the known pro-inflammatory factors IL1β, TNFα and PMA, or repressed by the anti-inflammatory factor, Dexamethasone (Dex). To understand how the mechanism of RNA-mediated activation compares with these pathways, we treated A549 cells with these compounds alone or in combination with RNA12.

**Figure 6.**
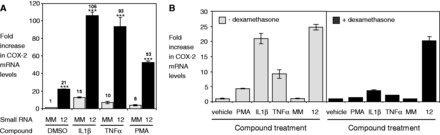
Small RNAs activate transcription through a distinct pathway. (**A**) qPCR data showing COX-2 mRNA expression following transfection (TRF) of either mismatched RNA (MM) or RNA12 (25 nM) in combination with either vehicle control, or activators of *COX-2* expression (IL1β, 10 ng/ml; TNFα, 100 ng/ml; PMA, 50 ng/ml). Numbers indicate fold activation relative to MM. Compound was added to cells 2 days after transfection of RNA and removed after 24 h. *n* = 3. (**B**) qPCR showing COX-2 mRNA levels following treatment with PMA (50 ng/ml), IL1β (10 ng/ml), TNFα (100 ng/ml) or RNA12 (25 nM) alone, or in combination with Dex (1 µM). Vehicle = control. Cells were treated with compound for 24 h. *n* = 2. Error bars are SD. ***P < 0.001 (*t*-test) relative to MM.

IL1β increased *COX-2* expression to a maximum level of 15–20-fold (Supplementary Figure S2D, Figure 6A), increased COX-2 pre-mRNA levels (Supplementary Figure S2B), and increased recruitment of RNAP2 at the *COX-2* promoter (Figure 5G). TNFα and PMA increased COX-2 levels by 10- and 8-fold, respectively (Figure 6A). Compared with treatment with IL1β, TNFα or PMA alone, when cells were treated cells with RNA12 in combination with IL1β, TNFα or PMA, we observed increased *COX-2* expression to higher levels, 106-, 93- and 53-fold, respectively (Figure 6A). Co-treatment with RNA12, therefore, yields expression levels that are much greater than can be achieved by the maximal doses of known activators of *COX-2*.

We also tested RNA12 in combination with Dex an anti-inflammatory agent known to repress *COX-2* expression. Addition of Dex reversed the effects of known activators PMA, IL1β and TNFα (Figure 6B). RNA12, by contrast, overrides repression by Dex.

Super-activation of *COX-2* expression beyond what can be induced by known activators, combined with the unique ability to override repression by Dex, indicates that the mechanism of promoter RNA-mediated activation is distinct from the known mechanisms of other inflammatory pathway regulators. Small RNAs provide a new sequence-specific pathway for activating *COX-2* expression that requires complementarity to the COX-2 promoter RNA.

### Sense promoter RNA is the molecular target for *COX-2* gene activation

Only one strand of miR-589 is complementary to the promoter RNA, making the sense strand the only potential partner for gene activation. RNA12, however, is complementary to both sense and antisense promoter RNAs making it possible that either stand might be its target.

**Figure 7.**
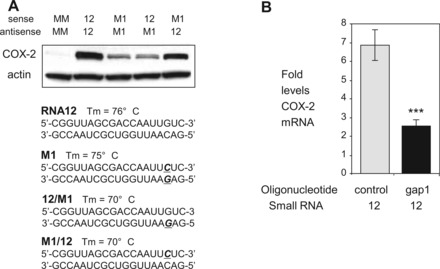
The sense promoter transcript is the target of RNA12. (**A**) Western showing effect of seed sequence mismatches on RNA-mediated activation of COX-2 protein expression. Mismatches are shown in bold/italicized/underlined. M1 duplex contains a single mismatch base in both strands. Sense strand is complementary to antisense promoter RNA. Antisense strand is complementary to sense promoter RNA. All duplex RNAs were transfected at 25 nM. (**B**) Effect of reducing sense promoter RNA using a gapmer oligonucleotide on induction of *COX-2* expression by RNA12. qPCR showing levels of COX-2 mRNA following initial transfection (TRF-1) with gapmer (gap1, 30 nM) to reduce sense RNA (−81/+56) and a second transfection (TRF-2) with RNA12 (12 nM). Control is a single-stranded gapmer oligonucleotide with no complementarity to *COX-2* RNA. *n* = 2. Error bars are SD. ***P < 0.001 (*t*-test) relative to MM.

To determine whether RNA12, like miR-589, targets the sense strand, we mutated a single base within the seed sequence on either strand of RNA12. We annealed the strands containing a mutation with native complementary strands lacking a mutation (Figure 7A). When the strand complementary to the sense promoter RNA was mutated at position 3 in the seed sequence, gene activation was almost abolished. Mutation of the strand complementary to the antisense strand had little impact. These data suggest that the sense strand of the promoter RNA is the molecular target for RNA-mediated activation of *COX-2* expression.

We also used antisense oligonucleotide gap1 to reduce expression of the sense RNA target and then treated cells with RNA12. Antisense gapmer oligonucleotides reduce promoter sense transcripts by inducing degradation of RNA-DNA targets through recruitment of RNase H. Because gapmers are single-stranded, they are good tools for distinguishing differences between the sense and antisense transcripts.

When RNA12 is introduced following transfection of A549 cells with gap1, we observed decreased activation of *COX-2* expression from 7- to 2.5-fold (Figure 7B). This result is consistent with promoter RNA acting as a scaffold that is necessary for small RNA-mediated molecular recognition and subsequent activation of *COX-2* expression. By reducing sense transcript levels, the gapmer removes many of the target sense transcripts for subsequent recognition by AGO2/small RNA complexes and would therefore block gene activation.

Taken together, three lines of evidence support the conclusion that *COX-2* gene activation by either RNA12 or miR-589 involves recognition of the sense promoter RNA, (i) miR-589 is complementary to the sense promoter transcript, not the antisense transcript; (ii) a single mutation of the seed sequence within the antisense strand of RNA12 blocks gene activation; and (iii) antisense oligonucleotide-mediated reduction of sense strand expression reverses gene activation.

### Promoter RNA acts as a scaffold for small RNA/RNAi factor complexes

**Figure 8.**
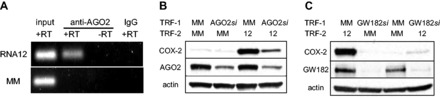
Gene activation by fully complementary RNA involves recruitment of RNAi factors. (**A**) RIP showing recruitment of AGO2 protein to promoter RNA by RNA12 (25 nM). (**B**) Western showing effect of reducing cellular AGO2 protein levels on COX-2 protein upregulation by RNA12 (25 nM). AGO2*si* is a pool of four duplex RNAs (12 nM) complementary to AGO2 mRNA. (**C**) Western showing effect of siRNA-mediated depletion of GW182 on upregulation of COX-2 protein by RNA12 (25 nM). GW182*si* is a duplex RNA pool (12 nM) complementary to mRNAs of TNRC6 paralogs.

To further explore the potential for mechanistic similarity between miR-589 and RNA12, we examined the role of RNAi factors. Similar to miR-589, activation by RNA12 also involved RNAi factors. RIP using nuclear extracts revealed that RNA12 recruited AGO2 to COX-2 promoter RNA (Figure 8A). When AGO2 levels are reduced using an anti-AGO2 siRNA, promoter RNA-mediated gene activation by RNA12 is blocked (Figure 8B). When GW182 levels are reduced using a siRNA pool to silence all three TNRC6 paralogs, promoter RNA-mediated gene activation by RNA12 is abolished (Figure 8C, Supplementary Figure S5). This requirement for AGO2 and GW182 parallels the mechanism of miR-589 action described earlier in the text.

### Cleavage of the promoter RNA is not necessary for gene activation

**Figure 9.**
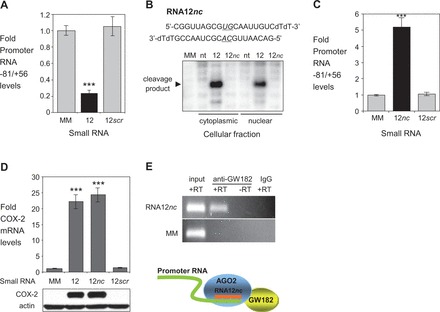
*COX-2* gene activation does not require cleavage of the promoter transcript. (**A**) qPCR showing effect of RNA12 or RNA duplex containing scrambled sequence (12*scr*) on promoter RNA (−81/+56) levels. *n* = 3. (**B**) *In vitro* analysis of target cleavage by RNA12 and RNA12*nc* (12*nc*). Bases within RNA12*nc* that are mismatched relative to the promoter RNA are underlined. (**C**) Effect of RNA12*nc* and RNA12*scr* on COX-2 promoter RNA levels (−81/+56). RNAs were transfected at 25 nM. *n* = 3. (**D**) qPCR and western showing effect of RNA12, RNA12*nc* and RNA12*scr* duplex on COX-2 mRNA levels (upper, *n* = 2) and protein levels (lower). RNAs were transfected at 25 nM. (**E**) RIP showing recruitment of GW182 to COX-2 RNA (−81/+56) by RNA12*nc* (25 nM). Scheme showing recruitment of AGO2 and GW182 proteins after addition of RNA12*nc* to an intact promoter RNA. Error bars are SD. ***P < 0.001 (*t*-test) relative to MM.

miR-589 and RNA12 share a requirement for RNAi factors. RNA12, however, is fully complementary and would be expected to induce AGO2-mediated cleavage of the promoter RNA (42). Consistent with this expectation, we observed that transfection of RNA12 into A549 cells reduces promoter RNA levels (Figure 9A). miR-589, by contrast, contains mismatches that disrupt the potential for cleavage by AGO2 (24) and does not reduce promoter RNA levels (Figure 3D).

To further investigate whether target cleavage was involved or required in the gene activation mechanism, we made RNA12 more ‘miR-like’ by introducing central mismatches to produce RNA12*nc* (*nc* = no cleavage) (Figure 9B). The two synthetic RNAs (RNA12 and RNA12*nc*) were transfected into cells. Nuclear or cytoplasmic extracts were prepared and used to test cleavage of a radiolabeled substrate RNA containing the common target site for RNA12 and RNA12*nc*.

RNA12 induced cleavage of the RNA substrate, whereas RNA12*nc* did not. Consistent with its failure to cleave a synthetic substrate and in contrast to RNA12 (Figure 9A), RNA12*nc* did not reduce promoter RNA levels (Figure 9C). Instead, similar to the effect on promoter RNA on addition of miR-589 (Figure 3D), addition of RNA12*nc* increased promoter RNA levels 5-fold.

RNA12 and RNA12*nc* have opposing effects on the level of promoter RNA, but their effects on *COX-2* expression and their mechanism of action appear to be the same. When added to A549 or T47D cells, RNA12*nc* activated *COX-2* expression as efficiently as RNA12 (Figures 5E and 9D). Like RNA12, RNA12*nc* super-activated *COX-2* expression when combined with IL1β, TNF or PMA (Supplementary Figure S2E). RIP using nuclear extracts demonstrated recruitment of GW182 to the promoter RNA (Figure 9E).

Our results showing that RNA12*nc* can induce activation of *COX-2* expression reinforce the data from miR-589 showing that activation can occur in the absence of target cleavage. These results further support the conclusion that RNA at the *COX-2* promoter acts as a scaffold for binding the small RNA/RNAi factor complexes that trigger gene activation. After induction of this gene activation cascade, the potential for cleavage of the promoter RNA and its absolute levels are not critical to mechanism.

### Gene activation involves modification of histones via WDR5

**Figure 10.**
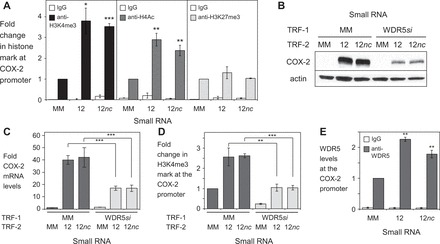
*COX-2* gene activation leads to histone modifications and requires expression of WDR5. (**A**) ChIP showing changes in H3K4me3 or H4Ac modifications at the *COX-2* promoter (site 8: −1540/−1464). Cells were treated with either mismatched control (MM), RNA12, or RNA12*nc* at 25 nM. RNAs were transfected at 25 nM. *n* = 3. See also Supplementary Figures S6A–D. (**B, C**) Western showing effect of siRNA-mediated silencing of WDR5 on induction of COX-2 protein (B) and mRNA (C) expression by RNA12 and RNA12*nc*. Cells were first transfected (TRF-1) with either mismatched control (MM) or anti-WDR5 siRNA then transfected with MM, RNA12 or RNA12*nc*. RNAs were transfected at 25 nM. (**C**) qPCR showing siRNA-mediated depletion of WDR5 mRNA. (**D**) ChIP showing changes in H3K4me3 modification at the *COX-2* promoter (site 8: −1540/−1464). Cells were first transfected (TRF-1) with either mismatched control (MM) or WDR5 siRNA (WDR5*si*) then transfected with MM, RNA12 or RNA12*nc*. RNAs were transfected at 25 nM. *n* = 3. (**E**) ChIP showing recruitment of WDR5 at the COX-2 promoter upon addition of RNA12 or RNA12*nc* (25 nM). (site 1: −81/+56). MM = mismatch control. Error bars are SD. *P < 0.05, **P < 0.01 and ***P < 0.001 (*t*-test) relative to mismatch control. All experiments were done in A549 cells.

To test the effects of promoter RNA-mediated gene activation on histone modification, we performed ChIP using primer sets covering regions from the +1 transcription start site to −1540 nucleotides upstream (Supplementary Figure S6A). H3K4 trimethylation and H4 acetylation are associated with gene activation. ChIP analysis using purified nuclear extract revealed both histone marks were increased following treatment with RNA12 or RNA12*nc* (Figure 10A, Supplementary Figure S6B and C). The occurrence of repressive mark H3K27 trimethylation was unchanged (Figure 10A, Supplementary Figure S6D).

WDR5 is a WD40 repeat-containing protein that can act as a protein scaffold to stimulate histone methyltransferase activity (43). WDR5 has been reported to associate with HOTTIP, an lncRNA that controls activation of several HOXA genes (44). To determine whether WDR5 was involved in RNA-mediated control of *COX-2* gene expression, we used an siRNA against WDR5 mRNA to decrease its expression (Supplementary Figure S6E).

We found that reduced levels of WDR5 blocked RNA-mediated gene activation of both COX-2 protein and mRNA expression by RNA12 and RNA12*nc* (Figure 10B and C). Reduced levels of WDR5 lowered the induction of H3K4me3 modification of histones by RNA12 and RNA12*nc* in purified nuclear extracts (Figure 10D). Also consistent with RNA-mediated action of WDR5, ChIP from nuclear extracts indicated that addition of RNA12 increased levels of WDR5 recruitment at the *COX-2* promoter (Figure 10E). These results suggest that RNA-mediated gene activation by small RNA/RNAi factor complexes induces chromatin modification and requires WDR5.

### Promoter RNA links activation of *COX-2* and *PLA2G4A* gene transcription

**Figure 11.**
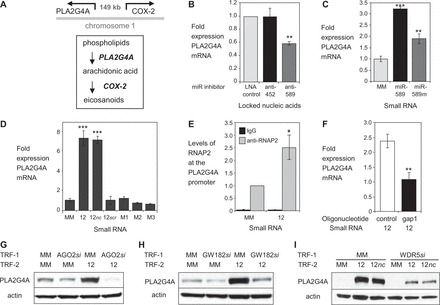
Promoter RNAs link *COX-2* and *PLA2G4A* transcription. (**A**) Distal and functional relationship of *COX-2* and *PLA2G4A*. Scheme showing the location and orientation of the *COX-2* and *PLA2G4A* promoters on chromosome 1 (top). Scheme showing the functional relationship between *COX-2* and *PLA2G4A* within the eicosanoid signaling pathway (bottom). (**B**) qPCR of PLA2G4A mRNA levels after transfection of anti-miRs (42 nM) that target either miR-452 or miR-589. *n* = 3. (**C**) qPCR of PLA2G4A mRNA after transfection of mature miR-589 or miR-589 mimic (miR-589 *m*) (42 nM). *n* = 2. (**D**) qPCR showing effect of RNA12, RNA12*nc*, RNA12 or duplex RNAs (25 nM) that contain scrambled (12*scr*) or mismatched bases (M1, M2, M3) on expression of PLA2G4A mRNA. *n* = 2. (**E**) ChIP showing recruitment of RNAP2 at the *PLA2G4A* promoter following transfection with RNA12 (25 nM). *n* = 4. (**F**) qPCR showing effect of reducing promoter RNA (−81/+56) on induction of *PLA2G4A* mRNA by RNA12. Cells were initially transfected (TRF-1) with gapmer oligonucleotide complementary to sense RNA (gap1, 30 nM) followed by a second transfection (TRF-2) with RNA12 (25 nM). Control is a non-complementary gapmer oligonucleotide. *n* = 2. (**G**) Western showing effect of reducing cellular AGO2 protein levels on PLA2G4A protein upregulation by RNA12 (25 nM). AGO2*si* (12 nM) is a pool of four duplex RNAs complementary to AGO2 mRNA. Cells were first transfected (TRF-1) with mismatched control (MM) or AGO2*si* then transfected with MM or RNA12. (**H**) Western showing effect of siRNA-mediated depletion of GW182 on PLA2G4A protein levels by RNA12 (25 nM). GW182*si* (12 nM) is a pool of duplex RNAs complementary to mRNAs of TNRC6 paralogs. Cells were first transfected (TRF-1) with mismatched control (MM) or GW182*si* then transfected (TRF-2) with MM or RNA12. (**I**) Western showing effect of siRNA-mediated depletion of WDR5 on induction of PLA2G4A protein expression by RNA12 or RNA12*nc* (25 nM). WDR5*si* (25 nM) is a duplex RNA complementary to WDR5 mRNA. Cells were first transfected (TRF-1) with mismatched control (MM) or WDR5*si* then transfected (TRF-2) with MM, RNA12 or RNA12*nc*. *n* = 4. Error bars are SD. *P < 0.05, **P < 0.01 and ***P < 0.001 (*t*-test) relative to mismatch control.

COX-2 is one of several key enzymes in the eicosanoid pathway that act in concert to regulate inflammation. PLA2G4A catalyzes the hydrolysis of membrane phospholipids to release arachidonic acid, the physiologic substrate of COX-2 (Figure 11A). The gene encoding PLA2G4A protein is adjacent to the *COX-2* gene on chromosome 1 and is ∼149 kb distant, indicating that the close functional partnership between the two genes may be accompanied by spatial proximity.

The physiologic connection and proximal relationship between *COX-2* and *PLA2G4A* led us to test the hypothesis that activating RNAs might also control *PLA2G4A* expression. Addition of the anti-miR targeted to miR-589 decreased basal *PLA2G4A* expression (Figure 11B). Conversely, addition of miR-589 led to an increase in basal expression levels (Figure 11C). RNA12 and RNA12*nc* also activated *PLA2G4A* expression, once again showing their functional equivalence (Figure 11D). As observed for activation of *COX-2* expression, insertion of seed sequence mismatches abolished activation of *PLA2G4A* expression. ChIP analysis from nuclear extracts revealed increased recruitment of RNAP2 at the *PLA2G4A* promoter, a result consistent with transcriptional activation by small RNA (Figure 11E).

Two conclusions can be drawn from these results. The first is that small RNAs that sequence-selectively activate *COX-2* expression also activate expression of *PLA2G4A*, the gene responsible for the enzyme that yields the substrate for the COX-2 enzyme. Activation of *PLA2G4A* mirrored activation of *COX-2*. The second conclusion is that a common mechanism of RNA-mediated regulation exists for the two genes.

Similarities in the mechanism of activation for *PLA2G4A* and *COX-2* include the following: (i) addition of antisense oligonucleotide gap1 to reduce levels of the sense promoter RNA reversed *PLA2G4A* activation by RNA12 (Figure 11F); (ii) siRNA-mediated reduction of AGO2, GW182 or WDR5 reversed activation of *PLA2G4A* expression (Figures 11GHI, Supplementary Figure S7A); (iii) addition of small RNA increased levels of H3K4me3 modifications throughout the *PLA2G4A* promoter, consistent with involvement of WDR5 in RNA-mediated activation (Supplementary Figure S7B and C); and (v) activation increased H4Ac marks across the *PLA2G4A* promoter (Supplementary Figure S7B and D). These mechanistic similarities suggest a link between the two genes that can be exploited by RNA.

It was possible that increased levels of COX-2 protein or enzymatic activity might explain increased expression of *PLA2G4A*. To test this hypothesis, we treated A549 cells with activators IL1β, TNFα or PMA to elicit maximal activation of COX-2 protein expression and observed a less than 2-fold increase in expression of *PLA2G4A* (Supplementary Figure S7E). Conversely, blocking COX-2 enzymatic activity by addition of inhibitor did not alter RNA-mediated activation of *COX-2* (Supplementary Figure S8A) or *PLA2G4A* (Supplementary Figure S8B).

### Physical linkage between *COX-2* and *PLA2G4A* promoters

One explanation for increased expression of both *COX-2* and *PLA2G4A* would be a physical linkage between the two genes. Gene looping can occur within a gene, between a gene and an enhancer site or between genes (45) to facilitate the action of noncoding RNAs (36). The *COX-2* and *PLA2G4A* gene loci map to chromosome 1q25 in a head to head configuration separated by 149 kb. There is promoter RNA overlapping the *COX-2* promoter, but no RNA was detected overlapping the *PLA2G4A* promoter in the RNA-Seq analysis. Gene looping would explain why interactions at the COX-2 promoter RNA might affect *PLA2G4A*.

To determine whether *COX-2* and *PLA2G4A* loci associate, we performed chromosome conformation capture (3C) analysis (46). In 3C, chromosomal DNA is cross-linked and treated with a restriction enzyme. Sequences that are held in close proximity by chromosomal structure, but which might be far distant from one another on the linear chromosome, can be ligated and subsequently detected by PCR and DNA sequencing.

**Figure 12.**
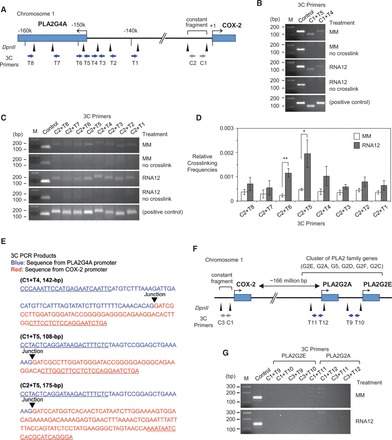
3C analysis indicates contacts between the *PLA2G4A* and *COX-2* promoters. The 3C experiments were performed using mismatch (MM)- or RNA12-treated sample (25 nM). (**A**) Diagram of *COX-2/PLA2G4A* loci and their intergenic region. Restriction enzyme *Dpn*II was used to digest chromosomal DNAs. Restriction sites for *Dpn*II and location of 3C primers are depicted by triangles and arrows, respectively. The 3C PCRs were performed using combinations of a constant primer (C1 or C2) and test primers (T1–8). Primer C1 and C2 are located within the constant fragment (−1077 to −354), which contains the *COX-2* promoter sequence. Primers T4, T5 and T6 target the *PLA2G4A* promoter (∼149k bases). (**B**) Analysis of 3C PCR products on 2.5% agarose gels (C1 + T4, C1 + T5). The internal region of the constant fragment was amplified using primer set −740/−600 as a control. *In vitro*-synthesized DNA templates that have sequences of expected ligation products were used as positive control and samples without cross-linking were used as negative control. (**C**) Analysis of 3C PCR products on 2.5% agarose gels (C2 + T1–8). The internal region of the constant fragment was amplified using primer set −740/−600 as a control. *In vitro*-synthesized DNA templates that have sequences of expected ligation products were used as positive control and samples without cross-linking were used as negative control. (**D**) Quantitative analysis of relative cross-linking frequencies using PrimeTime qPCR assay. *n* = 4. Error bars are SEM. *P < 0.05 and **P < 0.01 (*t*-test) relative to MM. (**E**) DNA sequencing of amplified 3C PCR products (C1 + T4, C1 + T5, C2 + T5) demonstrates a junction between the PLA2G4A and COX-2 promoter sequences. (**F**) Diagram of *COX-2/PLA2G2E/PLA2G2A* gene loci and design of 3C experiments. The 3C PCRs were performed using combinations of constant primers (C1 or C3) and test primers [T9, T10 (*PLA2G2E* promoter); T11, T12 (*PLA2G2A* promoter)]. Mismatch control or RNA12 were transfected at 25 nM. (**G**) Analysis of 3C PCR products on 2.5% agarose gels. Expected amplicon sizes for each primer set were 168-bp (C1 + T9), 131-bp (C1 + T10), 142-bp (C3 + T9), 105-bp (C3 + T10), 167-bp (C1 + T11), 165-bp (C1 + T12), 141-bp (C3 + T11) and 139-bp (C3 + T12), but no specific products were detected after 40 cycles of PCR amplification.

We performed 3C analysis with one primer (C1) at the *COX-2* promoter and other primers targeting the *PLA2G4A* promoter (primers T4 or T5) (Figure 12A, Supplementary Table S1). All experiments used purified A549 nuclei. Using SYBR-based PCR, a method that allows for the qualitative detection of cross-linked products, we observed amplified products in samples that had been cross-linked, but not in samples that were not cross-linked (Figure 12B).

The 5′-nuclease assays including PrimeTime (IDT) and TaqMan (ABI) qPCR are more suitable than the SYBR-based qPCR for 3C because a target-specific fluorescent probe for detection supplements gene-specific primers used to initiate product amplification. Use of a fluorescent probe for detection reduces the likelihood of detecting non-specific products and makes quantitative determinations more accurate. Using primers (C2 and T1–8) targeting the regions between *COX-2* and *PLA2G4A* genes and a probe for qPCR (Figure 12A), we determined whether associations were being selectively enriched near the transcription start sites for *COX-2* and *PLA2G4A*.

As observed when using SYBR chemistry, cross-linked products could be observed in the presence, but not in the absence, of cross-linking (Figure 12C). Quantitative measurement of amplification revealed that RNA12 selectively increased association between the *COX-2* and *PLA2G4A* promoters (Figure 12D). These results are consistent with the suggestion that RNA-mediated activation of *COX-2* and *PLA2G4A* by RNA12 is associated with closer proximity between the two promoters.

Sequencing the isolated ligation products confirmed junctions between the *COX-2* and *PLA2G4A* promoters (Figure 12E), indicating that the two regions were in close proximity in chromosomal DNA. The close physical proximity of the *COX-2* and *PLA2G4A* promoters provides a mechanism for communicating activation signals over long genomic distances and allows the functional partnership of the two enzymes during eicosanoid biosynthesis to be accompanied by linked expression.

*PLA2G4A* is just one of several *PLA2* family genes. We examined whether the *COX-2* promoter might also be physically linked to these related genes. 3C analysis with multiple primer sets (C1 or C3; T9–12) showed no evidence for connections between the *COX-2* promoter and genes encoding extracellular *PLA2* that cluster on the opposite arm of chromosome 1, ∼166 million bases distant (Figures 12FG).

## DISCUSSION

### RNAi factors interact with RNA in cell nuclei to activate gene expression

**Figure 13.**
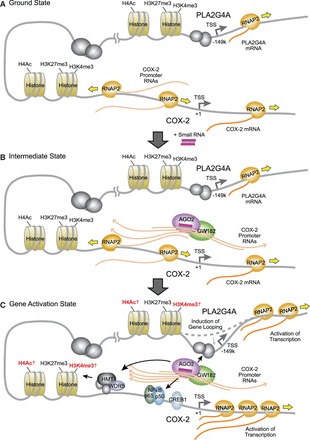
Model of promoter RNA-mediated activation of *COX-2* and *PLA2G4A* genes. (**A**) Ground state: promoter RNAs are expressed at the *COX-2* promoter. *COX-2* and *PLA2G4A* are expressed at low levels. (**B**) Intermediate state: The sense promoter RNA is a platform for recognition by a small complementary RNA (endogenous or synthetic) and recruitment of AGO2 and GW182. (**C**) Gene activation state: Binding of small RNA/AGO2/GW182 complex leads to association of transcription factors and WDR5, and an increase in activating histone marks. Specific gene looping is induced between the *COX-2* and *PLA2G4A* promoters, enabling activations of both genes.

miRNAs and RNAi factors are present in mammalian cell nuclei. These factors can form interactions with each other and with RNAs that overlap gene promoters (Figure 13A). Our results are best explained by a mechanism involving the RNA at the *COX-2* promoter acting as a scaffold for small RNA-mediated recruitment of AGO2 and GW182 near the *COX-2* promoter in cis (Figure 13B). Similar involvement of RNAi factors and nascent RNA scaffolds during transcriptional modulation and heterochromatin assembly in yeast has been noted (47,48). Recognition of the promoter RNA by the RNA-AGO2-GW182 complex may trigger recruitment of other factors like WDR5 and activation of *COX-2* and *PLA2G4A* gene expression. The 3C analysis reveals interaction between the *COX-2* and *PLA2G4A* promoters, demonstrating a gene architecture that permits long distance signaling (Figure 13C).

### Activation of *COX-2* expression requires on-target recognition at the *COX-2* loci

Whenever nucleic acids are introduced into cells there is a potential to produce off-target effects. These effects can confound experiments by causing misleading changes in target gene expression. Guarding against off-target effects is essential for any experiment with duplex RNA.

The use of multiple mismatched or scrambled control duplexes is a convenient and effective method to establish whether gene activation requires complementarity. We find that as little as one mismatch within the seed sequence largely abolishes gene activation (Figure 7A), consistent with an on-target effect that functions through the RNAi pathway. Scrambled sequences or sequences with multiple mismatches spread throughout the RNA were also inactive.

When RNA is introduced into cells, it can induce an interferon response that might be especially problematic for a highly inducible gene like *COX-2*. We observe little or no increase in the expression of a panel of interferon responsive genes (Supplementary Figure S4B).

*COX-2* gene activation is also achieved in the presence of RNA duplexes that contain modified nucleosides that are designed to reduce off-target effects (Supplementary Figure S2C). Addition of siRNAs designed to reduce AGO2 or GW182 expression, or a gapmer oligonucleotide designed to reduce levels of the target promoter transcript, reduce *COX-2* expression rather than increase it as would be expected from an off-target effect that occurs after addition of exogenous nucleic acid.

RNA12/RNA12*nc* and miR-589 have adjacent target sites near the *COX-2* transcription start site (Figure 5A), consistent with their similar ability to activate COX-2 gene expression. These targets sites, however, have different seed sequence matches. The fact that recognition by duplex RNAs with different seed sequence complementarities causes the same effect on expression of both *PLA2G4A* and *COX-2* further supports the conclusion that the effect is on-target. RIP using purified nuclei establishes that both RNA12/RNA12*nc* and miR-589 associate with the target *COX-2* promoter RNA, supporting an on-target physical interaction. RNA12 and RNA12*nc* have different sequences and different potential to induce cleavage of complementary target sites. The finding that their effects on *COX-2* expression are virtually identical further supports the conclusion that the observed activation is on-target.

The details of RNA-mediated gene activation differ from activation by known activators like IL1β, TNFα and PMA (Figure 6A). These data rule out an off-target effect through known pathways for *COX-2* activation. There is no known direct link between *COX-2* and *PLA2G4A* expression. Any off-target cause for linked upregulation of *COX-2* and *PLA2G4A* would be unprecedented. Taken together, our data and the robustness of RNA-mediated gene activation of *COX-2* and *PLA2G4A* make a strong case for on-target gene activation.

### Scaffold recognition, not strand cleavage, triggers activation

Although miR-589, RNA12 and RNA12*nc* are complementary to adjacent target sites, the three RNAs have fundamental differences in their structures and potential for affecting gene expression. They bind different sequences and differ in their complementarity relative to the promoter RNA. By evaluating their differences and similarities, it is possible to draw several conclusions about mechanism.

miR-589 and RNA12*nc* contain central mismatched bases that prevent cleavage of target transcripts by AGO2. RNA12, by contrast, is fully complementary and enables AGO2 to cleave the target RNA (Figure 9B). Our experimental observations are consistent with predicted potentials for strand cleavage. RNA12 reduces levels of COX-2 promoter RNA (Figure 9A) and induces cleavage of target RNA (Figure 9B), as would be expected for a fully complementary RNA/AGO2 complex. miR-589 and RNA12*nc*, which both contain central mismatches, do not reduce levels of the promoter RNA (Figure 3D, Figure 9C) or induce cleavage (Figure 9B).

The potential for cleaving the target transcript is a fundamental difference between RNA12 and miR-589/RNA12*nc*. Despite this fundamental difference, however, their activities are strikingly similar. RNA12/RNA12*nc* and miR-589 (i) activate *COX-2* and *PLA2G4A* expression; (ii) require seed sequence complementarity for activation; (iii) recruit AGO2 to the promoter RNA, (iv) induce histone modifications; (v) have their activity reduced by an antisense gapmer oligonucleotide that targets the promoter transcript; and (vi) require AGO2 and GW182 expression for activity.

The functional similarity of miR-589, RNA12 and RNA12*nc*, despite their different potential to induce cleavage of target, demonstrates a central feature of mechanism. Cleavage of the target RNA is not required for gene activation. Instead, the initial recognition step of the *COX-2* promoter RNA by the small RNA and associated protein factors is the critical switch that triggers activation. The subsequent fate of the promoter RNA after it binds the RNA/AGO2 complex, cleaved or uncleaved, has little effect on activation. Our results suggest that the most likely role for the promoter RNA is to act as a scaffold.

Antisense oligonucleotide gap1 reduces levels of the promoter transcript by RNase H-mediated cleavage. Reduction of the promoter transcript by gap1 does not increase *COX-2* expression (Figure 3H), further supporting the conclusion that cleavage of the transcript is not necessary for activation. Indeed, we observe gap1 leads to a decrease of *COX-2* expression, consistent with the promoter transcript being needed as a scaffold during activation.

Both RNA12 and antisense oligonucleotide gap1 reduce promoter RNA levels (Figure 9A, Figure 3G), but only RNA12 causes activation. This may seem contradictory, but the contradiction is resolved when the different mechanisms of the two nucleic acids are considered. gap1 is an antisense oligonucleotide, not a duplex RNA. Because it is a single-stranded RNA-DNA hybrid, it does not recruit AGO2. By contrast, when RNA12 recognizes the promoter transcript, it recruits AGO2 and other factors such as GW182 (Figure 8A).

This AGO2/RNA complex recognition step, which gap1 does not share but which is shared by RNA12*nc* and miR-589, is the likely trigger for gene activation. When gap1 was added before RNA12, the addition of gap1 caused loss of the transcript before RNA12 can recruit AGO2. By removing the scaffold and the potential to recruit AGO2, activation is prevented. Activation requires an intact transcript for recognition and a small RNA capable of recruiting AGO2 and associated protein factors to the target.

### Promoter RNAs are targets for ribonucleoprotein transcription factors

RNAs that overlap gene promoters could influence gene expression through several different mechanisms. These include the following: (i) transcriptional interference caused when synthesis of the lncRNA interferes with transcription of mRNA; (ii) direct hybridization between an antisense transcript and an mRNA; (iii) miRNAs or other functional RNAs encoded within lncRNAs; and (iv) global recruitment of histone or DNA modifications. All of these mechanisms may be physiologically relevant in mammalian cells, but our results with *COX-2/PLA2G4A* activation through promoter RNA recognition demonstrate a novel mechanism—small RNA-dependent recruitment of factors at a promoter in *cis* to affect the protein machinery and the transcription of a specific gene.

Efficient RNA recognition in the nucleus might have advantages as a mechanism for control of splicing or transcription. Although proteins are powerful agents for controlling expression, they lack a general capacity to be highly selective for just one gene or to rapidly evolve specificities for recognizing new genes. Because lncRNAs, miRNAs, AGO2 and GW182 exist in the nucleus, they are the basis for an alternate regulatory pathway that exploits the versatility of Watson–Crick base-pairing. When AGO2 binds a small RNA, it forms a ribonucleoprotein complex and facilitates recognition of complementary targets within nuclear RNAs. Sequence-specific recognition using Watson–Crick base pairing can evolve new specificities relatively rapidly using conserved core RNA/protein machinery.

When lncRNAs are synthesized as nascent transcripts, they are close to gene promoters and poised to influence transcription in *cis*. Even if present at just a few copies per cell, the effective concentration of nascent transcripts relative to promoter DNA is high. Like a transcription factor binding to a promoter, or a ligand changing the conformation of a nuclear hormone receptor, delivery of the RNA/protein complex to the promoter RNA scaffold triggers gene activation. We have recently observed that the COX-2 promoter transcript is present at ∼2 copies per A549 lung cancer cell (22), consistent with action in *cis* but not consistent with the expectation that the transcript might locate its target in *trans*.

### Implications for regulation of COX-2

It seems logical that *PLA2G4A* and *COX-2* expression should be linked, as COX-2 enzyme uses the product of PLA2G4A enzyme. However, despite of the central role of eicosanoid signaling in inflammation and other critical physiologic processes, mechanisms for coordinating their expression have not been described. We find that *COX-2* and *PLA2G4A* are both induced by an RNA-mediated mechanism that can be triggered by endogenous miR-589. RNA provides a mechanism that allows coordinated expression of two key enzymes within the eicosanoid pathway.

RNA-mediated induction coordinates COX-2 and PLA2G4A protein levels selectively, unlike the more global gene induction triggered by IL1β and other known inducers. For example, *COX-1* and *COX-2* differ dramatically in their regulation. *COX-2* expression is usually induced while *COX-1* is constitutively expressed. Gene looping and recognition of the *COX-2* promoter RNA by miR-589/RNAi factor complex provide a means for coordinated expression of *COX-2*, but not *COX-1*, to PLA2G4A synthesis.

### Addressing an alternate hypothesis to explain COX-2/PLA2G4A activation

Any experiment that uses nucleic acids inside cells runs the risk of inducing off-target effects. As noted earlier in the text, we have performed several experiments to minimize this possibility. One alternate explanation, however, would be if miR-589 and the other five activating RNAs were modulating expression of one or more transcription factors, which in turn increased both *COX-2* and *PLA2G4A* expression.

We believe that this is unlikely for the following reasons: (i) As miR-589, RNA12, RNA12*nc*, RNA10, RNA13 and RNA38 all activate COX-2 expression, they would all need to target a transcription factor that controls both *COX-2* and *PLA2G4A*; (ii) None of the RNA activators have seed complementary to known transcriptional repressors of *COX-2* or *PLA2G4A*; (iii) As activation by RNA in combination with well-established physiologic *COX-2* activators is additive, the putative transcription factor would need to control a new pathway for *COX-2* activation; (iv) An oligonucleotide gapmer that reduces expression of the target promoter transcript also reduces gene activation by miR-589 and RNA12. If activation was being modulated through downregulation of a transcription factor, targeting the promoter transcript would be expected to have no effect; and (v) We observe physical interactions between the promoter transcript and AGO2. We also observe interactions between the promoters for *PLA2G4A* and *COX-2*. These contacts describe direct physical interactions between the major factors involved in our proposed mechanism of action, reducing the need to invoke an indirect mechanism that involves unidentified transcription factors.

It could also be argued that the small RNAs are titrating a repressor that normally binds to the *COX-2* and *PLA2G4A* promoters. For such binding to affect gene expression in our experiments, it would need to be acutely sensitive to just a single change to one nucleoside of the duplex within the region normally associated with seed sequence activity for RNAi because one substitution abolishes activation (Figure 7A) or to the introduction of other mismatched bases (Figure 5D, Figure 11D). The repressor titration would need to be insensitive to introduction of two mismatched bases (activation is still achieved by RNA12nc) or more extensive disruption of base-pairing (activation by incompletely matched miR-589). The repressor would also need to be titrated by two families of RNAs (miR-589 and RNA12/RNA12*nc*/RNA10/RNA13/RNA38) that share little overlap in their sequences. The repressor would need to control both *PLA2G4A* and *COX-2*, even though the promoters share no recognized repressor binding sites. Repressors normally bind B-form DNA, but for titration to occur in this case they would need to recognize a short RNA that can be chemically modified (Supplementary Figure 2C). The duplex RNA would also need to survive inside mammalian cells without being degraded or loaded into the RISC complex as single strand. Finally, a mechanism of inactivation that involves titration of a repressor would be inconsistent with dependence on AGO2 or GW182 or the observed reversal of activation on addition of a gapmer targeting the promoter transcript.

Finally, we note that activation of *COX-2* by duplex RNAs that target promoter transcripts near the transcription start site is not a unique observation. Our laboratory has previously observed similar results with duplex RNAs targeting promoter regions for progesterone receptor (20,34,35) and LDL receptor (21). The activating RNAs responsible for controlling LDL receptor, progesterone receptor and *COX-2* expression have little sequence similarity, and regulate both TATA-less and TATA-box promoters. For all three gene targets, binding to a nuclear promoter transcript was observed. The common activating effects by RNAs that are complementary to different gene promoters support the conclusion that the ability to sequence-specifically recognize the gene target is critical for activity.

### Future directions for research

Our study is a step toward understanding the potential of RNAi factors to control gene expression in cell nuclei. Much remains to be learned. The presence of AGO2 and other RNAi factors in mammalian cell nuclei continues to be controversial despite significant experimental evidence that AGO2 can be a nuclear factor (26). It is essential that experiments be performed to move this debate forward.

Our work suggests that nuclear RNAi factors can be robust regulators of gene expression and that evolutionary pressure should lead to other genes being influenced by nuclear RNAi. Sequencing of RNAs bound to RNAi factors in cell nuclei may help identify likely gene targets for endogenous regulation. We have found that validating endogenous targets requires a painstaking and resource-intensive effort. Because the experiments are demanding, using RNA-seq to rank targets is a high priority. Experiments that systematically investigate potential endogenous targets would determine whether nuclear RNAi has broad potential for gene regulation and are needed to justify the investment required to pursue *in vivo* investigations.

Our studies are not intended to lay out a complete roadmap for how recognition of a promoter transcript by a small RNA in complex with RNAi factors can affect gene expression. As with protein transcription or splicing factors, a full inventory of influential partners and elucidation of a step-by-step mechanism will require more extensive research and may vary from one gene target to the next. Similarly, the importance of histone modification, whether it is a cause or consequence of gene activation, will be an important goal for future research into the molecular details of RNA-mediated gene activation.

We are less optimistic about the potential for using vector-based technologies as a tool for investigating physiologic systems that, like ours, appear to function in *cis*. For example, we previously observed that overexpression of a promoter transcript required for RNA-mediated gene activation had no effect on expression of the associated mRNA (36).

The value of vector-based systems for evaluating cytoplasmic miRNA interactions is well-established. In the nucleus, however, our data suggest a more complex interplay of promoter transcripts, RNAi factors, histone modifications and gene loci at the chromatin level. It seems unlikely that reporter constructs will often serve as good mimics of the endogenous system. Even if effects are observed, it is not clear to us that the considerable effort needed to set up a reporter system will lead to results that will be relevant to the parent gene. At a minimum, the disadvantages of moving away from studying the endogenous gene and the time needed to develop a reporter system must be carefully weighed against any perceived advantages that a reporter system might bring.

One possible exception would be site-specific mutagenesis of mammalian chromosomes. Such alterations can now be achieved with zinc fingers or TALEN technologies (49). While beyond the scope of our study, selective mutagenesis would be important for eliminating any uncertainty about the direct target for small RNAs that modulate transcription. These experiments, however, are not trivial. They would require that all chromosomes be mutated and that the mutations not disrupt existing transcription factor binding sites, such as the TATA box site adjacent to the miR-589 target at the *COX-2* promoter. As with the use of vector constructs, there exists a danger that altering the endogenous gene might yield results that apply only to the mutant construct.

We have now shown that small RNAs targeted to sequences near transcription start sites can upregulate expression of three loci, *COX-2*/*PLA2G4A*, progesterone receptor (20,34–37) and LDL receptor (21). Aside from any natural regulatory role for small RNAs in mammalian nuclei, these data suggest that it might be possible to upregulate therapeutic targets. This should be another goal for research.

Our experience suggests that the key step toward therapeutic upregulation is identifying a suitable gene target that justifies investing the considerable effort needed to find and validate activating RNAs. Once a compelling disease target is identified, pursuing experiments similar to those outlined in our report should permit a relatively rapid yes or no decision about whether RNA-mediated activation is feasible and also provide insights into the potential mechanism of action.

## CONCLUSION

Promoter RNA can act in conjunction with small RNAs and RNAi factors to organize a multigene network of transcriptional regulation and trigger enhanced expression. In contrast to miRNAs in the cytoplasm, which commonly induce modest changes in gene expression of >2–3-fold, we observe a dramatic increase of >25-fold, which can be enhanced to >100-fold when combined with other natural activation pathways.

Our results may be broadly relevant to mammalian gene regulation. miRNAs and RNAi factors GW182 and AGO2 are in the nucleus, providing the macromolecular machinery needed for sequence-specific recognition. Non-coding RNAs that are associated with chromatin offer a recognition point for RNA-mediated control of transcription at specific loci, making the concept a logical starting point for understanding the importance of nuclear RNA. RNA-mediated gene activation by both fully complementary duplex RNAs and partially complementary miR-589 is robust and potent, making it likely that the pathway would be exploited during evolution (26). miR-589 is conserved in primates, as are its target sites within the *COX-2* promoter and the spatial organization of the *COX-2* and *PLA2G4A* genes.

Gene loops are relatively common at gene promoters and often extend beyond adjacent genes (50). Together, RNA-mediated recognition and *cis*-acting gene loops allow the *COX-2* and *PLA2G4A* loci to form a functional operon that can affect the efficiency of eicosanoid biosynthesis. If RNA-mediated transcription linkages are found to be general for other pairs of functionally related pathway genes, they would have far reaching implications as a regulatory mechanism for sequence-specifically controlling the transcription of gene networks. Small RNAs that are designed to recognize nascent RNAs can achieve potent and selective activation of single genes or gene networks and provide a starting point for a new class of therapeutic agent.

## SUPPLEMENTARY DATA

Supplemental Data is available at NAR Online.

## FUNDING

National Institutes of Health (NIH) [GM 73042/DRC, GM85080/BAJ]; Welch Foundation [I-1244/DRC]; Cancer Prevention and Research Institute of Texas [RP120311/BAJ]. Funding for open access charge: NIH.

*Conflict of interest statement*. None declared.

## Supplementary Material

Supplementary Data
