# Supplementary material for: Promoter RNA links transcriptional regulation of inflammatory pathway genes
Source: Nucleic Acids Res. 2013 Aug 31;41(22):10086–109. doi: 10.1093/nar/gkt777 (PMC3905862; doi:10.1093/nar/gkt777)
Supplement: Supplementary Data [file supp_41_22_10086__index.html]

Promoter RNA links transcriptional regulation of inflammatory pathway genes — Promoter RNA links transcriptional regulation of inflammatory pathway genes — Supplementary Data 

# Promoter RNA links transcriptional regulation of inflammatory pathway genes

## Supplementary Data

files

**Files in this Data Supplement:**

- Supplementary Data - pdf file
