# Supplementary material for: Promoter RNA links transcriptional regulation of inflammatory pathway genes
Source: Nucleic Acids Res. 2013 Aug 31;41(22):10086–109. doi: 10.1093/nar/gkt777 (PMC3905862; doi:10.1093/nar/gkt777)
Supplement: Supplementary Data [file supp_gkt777_nar-01987-h-2013-File004.pdf]

Supplementary Information

Promoter RNA Links Transcriptional Regulation of Inflammatory Pathway Genes

Masayuki Matsui§, Yongjun Chu§, Huiying Zhang, Keith T. Gagnon, Sarfraz Shaikh, Satya Kuchimanchi, Muthiah Manoharan, David R. Corey*, and Bethany A. Janowski*

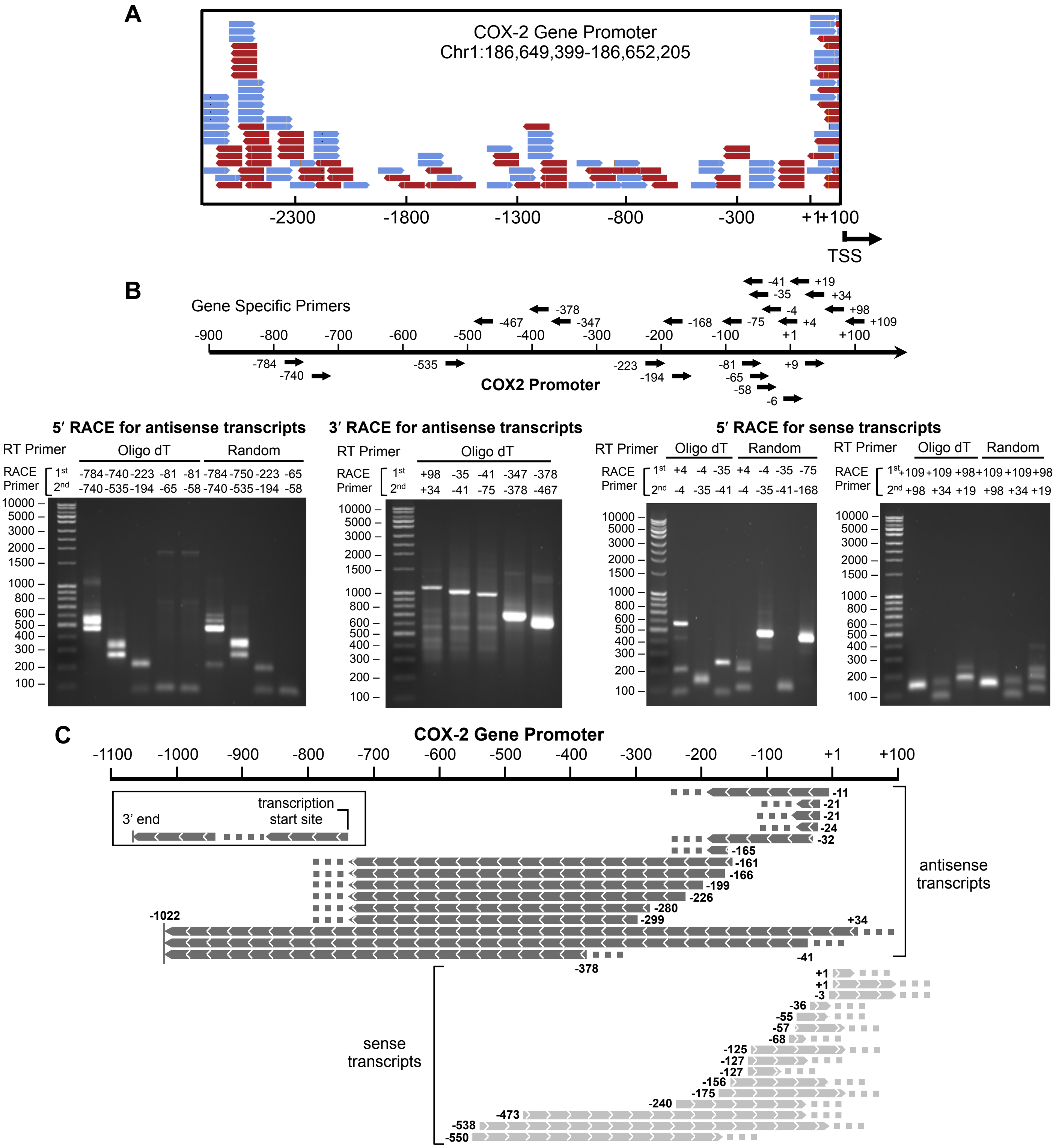

Supplementary Figure S1. Transcription at the COX-2 locus.
(A) Location of RNA sequence reads uniquely mapped to the COX-2 promoter. The paired-end reads in red color are in the anti-sense direction and blue color in sense direction relative to COX-2 mRNA direction. The sequencing data is not strand-specific. (B) Gene specific primers used for 5' and 3' RACE (upper) and analysis of RACE PCR products on 1% agarose gels (lower). (C) Schematic showing transcription start sites and 3' ends identified by 5' and 3' RACE.

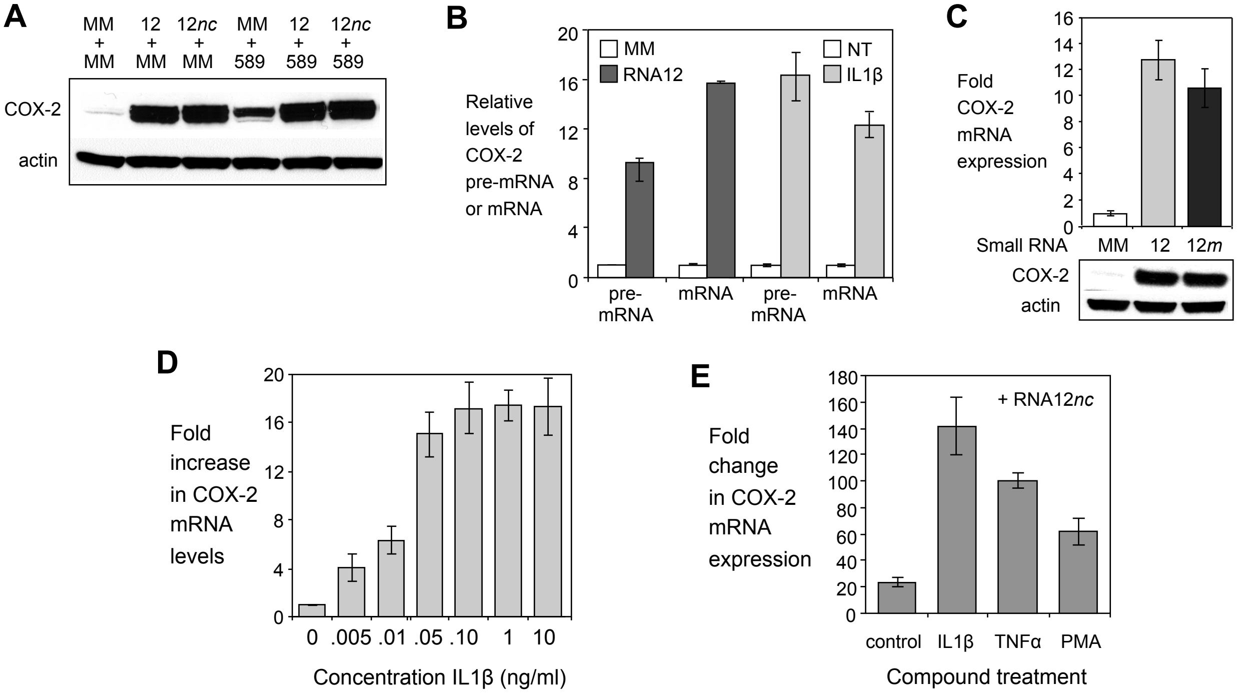

Supplementary Figure S2. COX-2 activation by small RNAs and compounds.
(A) Western showing effect of combining RNA12 (12 nM) or RNA12*nc* (12nM) with mature miR-589 (30 nM) on activation of COX-2 protein expression. (B) qPCR data showing increased expression of COX-2 pre-mRNA and mRNA following transfection with RNA12 or treatment with interleukin-1beta (IL1β, 10 ng/ml). MM=mismatched RNA control. RNA was transfected at 25 nM. NT=no treatment. (C) qPCR and western data showing increased COX-2 mRNA (upper) and protein (lower) by RNA12 (25 nM) and RNA12*m* (25 nM), an RNA with chemical modifications designed to reduced interferon responsiveness. (D) qPCR data showing the effect of increasing IL1β concentrations on COX-2 mRNA expression. (E) qPCR data showing the effect of combining RNA12*nc* (25 nM) with activators interleukin-1beta, (IL1β, 10 ng/ml), tumor necrosis factor-alpha (TNFα, 100 ng/ml), phorbol 12-myristate 13-acetate (PMA, 50 ng/ml). Cells were treated with compound two days after transfection of RNA12*nc* then removed after 24 h. All experiments were performed in A549 cells.

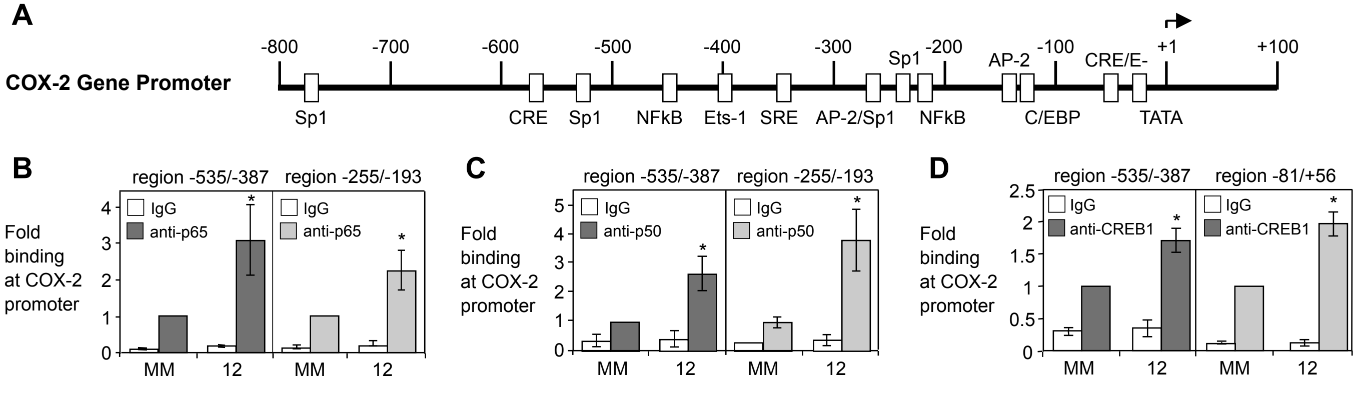

Supplementary Figure S3. Chromatin immunoprecipitation for transcription factors.
(A) Schematic showing COX-2 promoter and potential transcription factor binding sites. (B–D) Chromatin immunoprecipitation (ChIP) showing increased recruitment of transcription factors, NFkB (p65 (B), p50 (C)) and CREB1 (D) to the COX-2 promoter. Region amplified is designated above each data set. Cells were first transfected with either mismatched control (MM) or RNA12. RNAs were transfected at 25 nM. n=3.

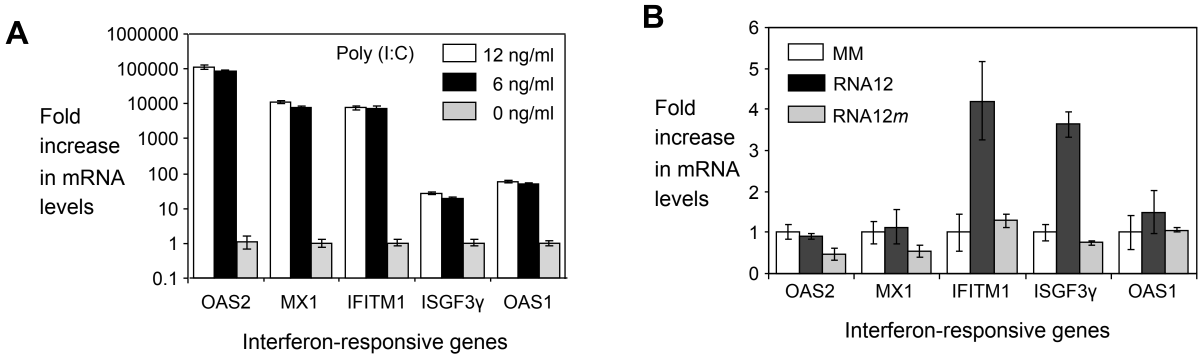

Supplementary Figure S4. Effect of treatment with poly (I:C) or activating RNAs on interferon responsive gene expressions.
(A) qPCR data showing effect of adding poly I:C on the mRNA expression of interferon responsive genes (log scale). (B) qPCR data showing the effect of RNA12 (25 nM) and RNA12m (25 nM) on mRNA expression of interferon responsive genes (linear scale). Experiments were performed in A549 cells. Error bars are SD.

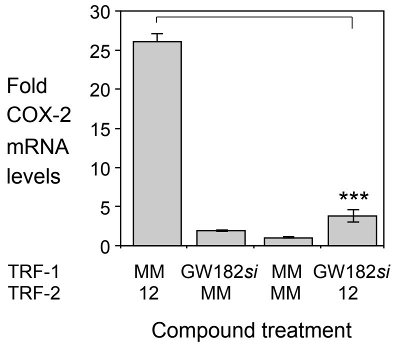

Supplementary Figure S5. COX-2 activation by RNA12 requires GW182.
qPCR data showing the effect of siRNA-mediated depletion of GW182 on induction of COX-2 mRNA expressions by RNA12 (25 nM). GW182*si* (12 nM) is a pool of duplex RNAs complementary to mRNAs of TNRC6 paralogs. A549 cells were first transfected (TRF-1) with mismatched RNA control (MM) or GW182*si* then transfected (TRF-2) with MM or RNA12. Error bars are SD. ***p < 0.001 (t-test) relative to mismatch control.

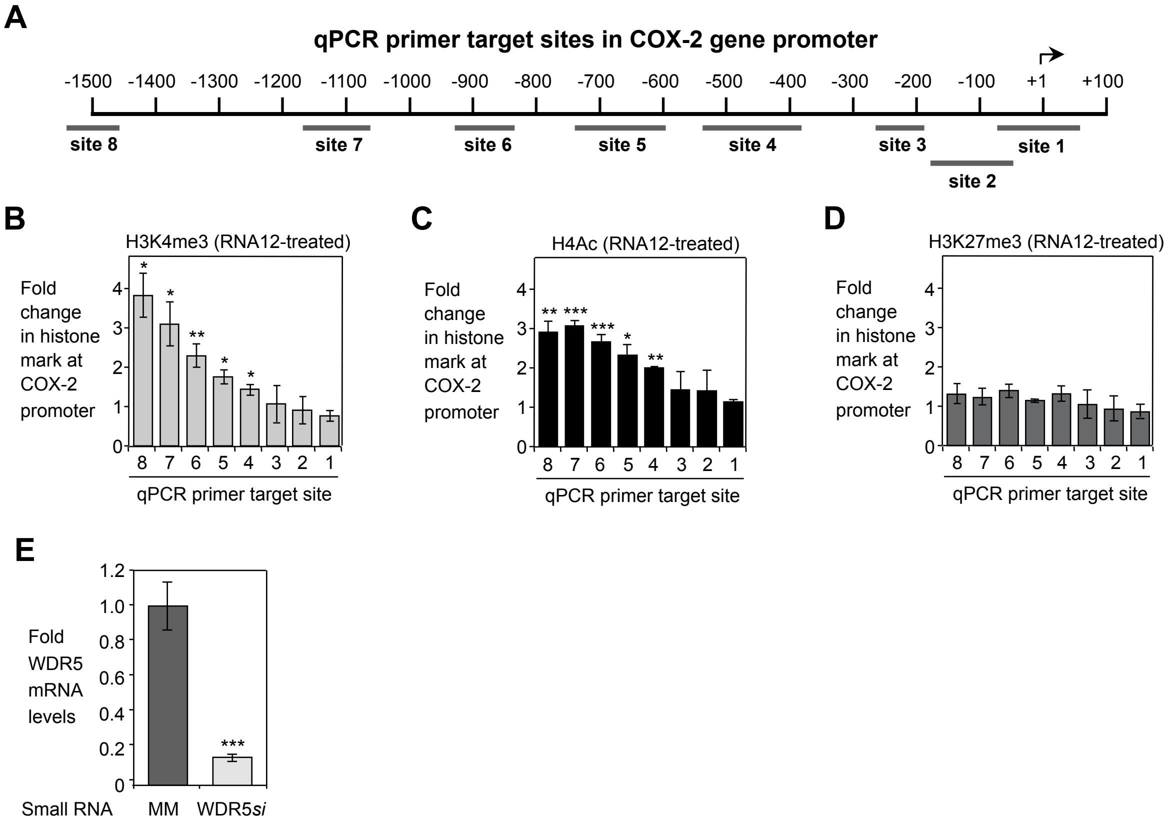

Supplementary Figure S6. Gene activation by small RNA involves histone methylation. (A) Schematic of target sites for qPCR primers used to examine histone modifications at the COX-2 promoter. (B–D) ChIP showing the effect of lncRNA-mediated COX-2 activation by RNA12 on H3K4me3, H4Ac, and H3K27me3 modifications. n=3–4. RNA12 or mismatched RNA control (MM) was transfected at 25 nM into A549 cells. (E) qPCR showing siRNA-mediated depletion of WDR5 mRNA. WDR5*si* (25 nM) is a duplex RNA complementary to WDR5 mRNA. Error bars are SD. *p < 0.05, **p < 0.01, and ***p < 0.001 (t-test) relative to mismatch control.

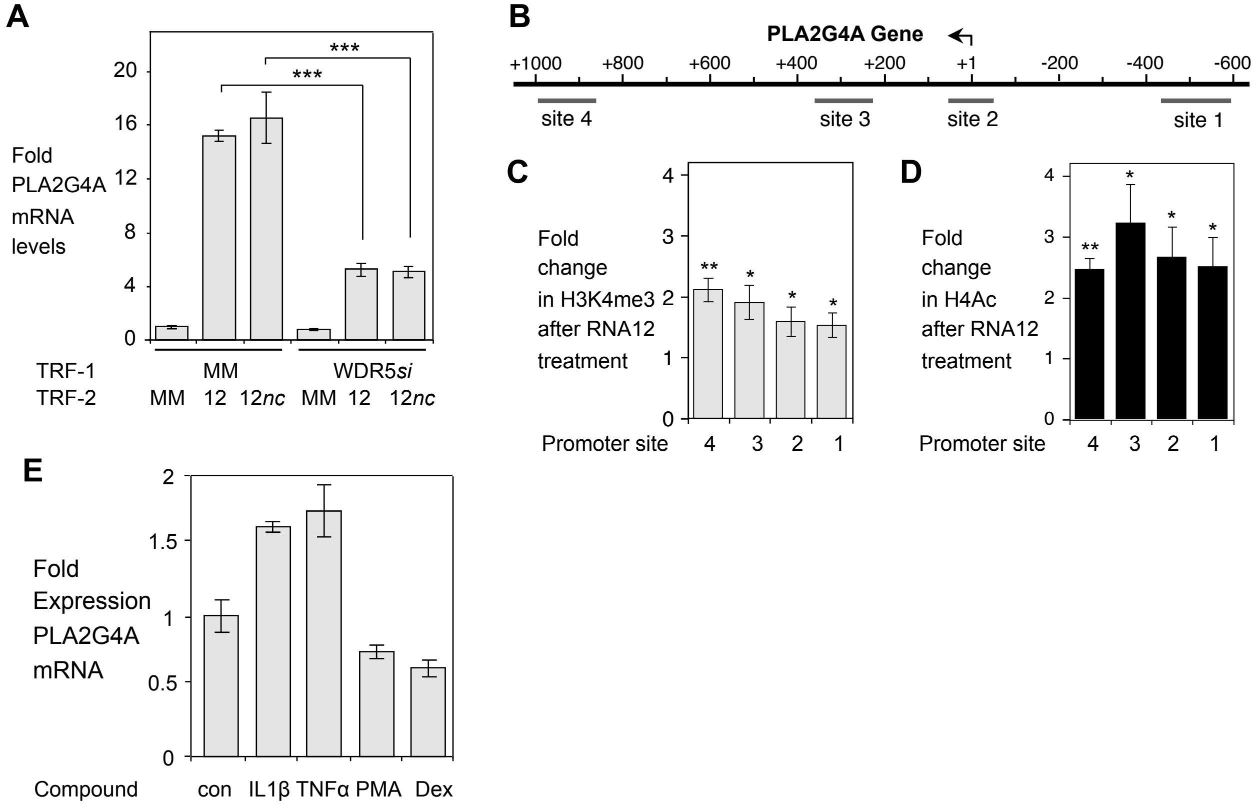

Supplementary Figure S7. Promoter RNAs link COX-2 and PLA2G4A transcription.
(A) qPCR showing effect of siRNA-mediated depletion of WDR5 on PLA2G4A mRNA expression in cells transfected with RNA12 or RNA12*nc*. WDR5*si* (25 nM) is a duplex RNA complementary to WDR5 mRNA. A549 cells were first transfected (TRF-1) with mismatched RNA control (MM) or WDR5*si* then transfected (TRF-2) with MM, RNA12, or RNA12*nc* at 25 nM. n=4. (B) Target sites for qPCR primers used to examine histone modifications. (C,D) ChIP showing changes in H3K4me3 (C) and H4Ac (D) modifications at the PLA2G4A promoter following transfection of RNA12 (25 nM). n=3. (E) qPCR data showing effect of activators IL1β (10 ng/ml), TNFα (100 ng/ml), PMA (50 ng/ml), or dexamethasone (Dex, 1 μM) on PLA2G4A mRNA expression. A549 cells were incubated with compound then removed 24 h later. Error bars are SD. *p < 0.05, **p < 0.01, and ***p < 0.001 (t-test) relative to mismatch control.

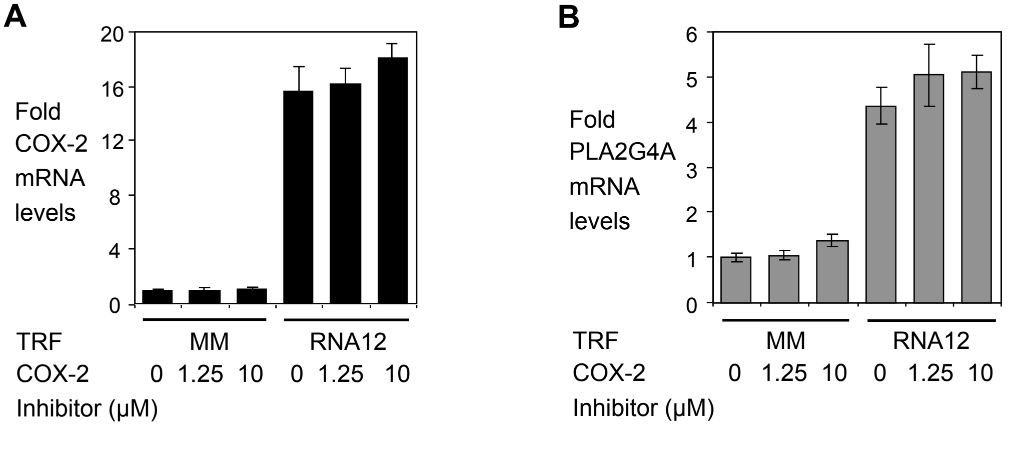

Supplementary Figure S8. Effect of COX-2 inhibitor on COX-2 or PLA2G4A activation by RNA12.
qPCR data showing that activation of COX-2 (A) or PLA2G4A (B) mRNA expression is not affected by addition of a COX-2 inhibitor (Cayman Chemicals, SC58125). The inhibitor is 150 times more potent in the inhibition of COX-2 than COX-1. Cells were first transfected (TRF) with mismatched RNA control (MM) or RNA12. Inhibitor was added two days after transfection then removed 24 h later. Error bars are SD.

Supplementary Table S1. Primers and a probe used in this research and their targets.

Name	Sequence	Assay used for	Target
Primer-1540F	CCGTGTCTCATGAAGAATCAG	ChIP	COX-2 Promoter
Primer-1464R	AAATAAGAGACTCACTTGGAGG	ChIP	COX-2 Promoter
Primer-1167F	ATTCCTCATCCAACTATGTTCC	ChIP	COX-2 Promoter
Primer-1063R	TTGTGACCATGGATCAAAGTAC	ChIP	COX-2 Promoter
Primer-925F	CGTTTTGGACATTTAGCGTC	ChIP	COX-2 Promoter
Primer-834R	CCACGGGTCACCAATATAAAG	ChIP	COX-2 Promoter
Primer-740F	GGGTGAAGGTACGGAGAACA	qPCR, ChIP	COX-2 Promoter/Promoter RNAs
Primer-600R	TGCAGCACATACATACATAGCTT	qPCR, ChIP	COX-2 Promoter/Promoter RNAs
Primer-535F	AACCTTACTCGCCCCAGTCT	qPCR, ChIP, RIP	COX-2 Promoter/Promoter RNAs
Primer-387R	GACACTTGGCTTCCTCTCCA	qPCR, ChIP, RIP	COX-2 Promoter/Promoter RNAs
Primer-255F	GGAGGAGAGGGAGGGATCAG	qPCR, ChIP	COX-2 Promoter/Promoter RNAs
Primer-193R	TGCCCCAATTTGGGAGC	qPCR, ChIP	COX-2 Promoter/Promoter RNAs
Primer-187F	CTGGGTTTCCGATTTTCTCA	qPCR	COX-2 Promoter/Promoter RNAs
Primer-81F	AAGGCGGAAAGAAACAGTCA	qPCR, ChIP, RIP, RACE	COX-2 Promoter/Promoter RNAs
Primer-48R	CCCATGTGACGAAATGACTG	qPCR	COX-2 Promoter RNAs
Primer-17F	TCTCTCGGTTAGCGACCAAT	qPCR	COX-2 Promoter RNAs
Primer+3R	ATTGGTCGCTAACCGAGAGA	RIP	COX-2 Promoter RNAs
Primer+5F	GTCATACGACTTGCAGTGAG	qPCR	COX-2 mRNA
Primer+26R	CGCTCACTGCAAGTCGTATG	RIP	COX-2 Promoter RNAs
Primer+56R	CTGCTGAGGAGTTCCTGGAC	qPCR, ChIP, RIP	COX-2 Promoter/Promoter RNAs
Primer+106R	GGGTAGGCTTTGCTGTCTGA	qPCR	COX-2 mRNA/Promoter RNAs
Primer exon1F	TCAGACAGCAAAGCCTACCC	qPCR	COX-2 mRNA
Primer exon2R	GTTTTGACATGGGTGGGAAC	qPCR	COX-2 mRNA
Primer exon2F	TGAGTGTGGGATTTGACCAG	qPCR	COX-2 mRNA
Primer exon3R	TGTGTTTGGAGTGGGTTTCA	qPCR	COX-2 mRNA
Primer exon10F	TTCCAGATCCAGAGCTCATTAAA	qPCR	COX-2 mRNA
Primer exon10R	CCGGAGCGGGAAGAACT	qPCR	COX-2 mRNA
Primer intron3F	GGTAGCATGGTCCAGCTGTT	qPCR	COX-2 pre-mRNA
Primer intron3R	GCTGAGTATGGCACCCACTT	qPCR	COX-2 pre-mRNA
PLA2G4A Fwd	ACTGCACAATGCCCTTTACC	qPCR	PLA2G4A mRNA
PLA2G4A Rev	CGGGAGCCATAAAAGTACCA	qPCR	PLA2G4A mRNA
GAPDH Fwd	TGGTATCGTGGAAGGACTCATGAC	qPCR	GAPDH mRNA
GAPDH Rev	ATGCCAGTGAGCTTCCCGTTCAGC	qPCR	GAPDH mRNA
PLA2G4A -594F	CACCTTCTTCATTACAGTGCTA	ChIP	PLA2G4A Promoter
PLA2G4A -426R	AGACTCAGAACACCTGTGTTG	ChIP	PLA2G4A Promoter

Supplementary Table S1. (Continued)

Name	Sequence	Assay used for	Target
PLA2G4A -48F	TTCTATGAGAAGAGAGCGTTC	ChIP	PLA2G4A Promoter
PLA2G4A +52R	AGTAGGAGGGGCTAAAATGTG	ChIP	PLA2G4A Promoter
PLA2G4A +235F	AGAAAGGACGGAGGGGAAAC	ChIP	PLA2G4A Gene
PLA2G4A +368R	CAGTGCATTTAGATCCAGGAAG	ChIP	PLA2G4A Gene
PLA2G4A +866F	AGTGGATGAATGTATCTGTCTCC	ChIP	PLA2G4A Gene
PLA2G4A +996R	CTGCCTTAGCTGAATAAATGC	ChIP	PLA2G4A Gene
WDR5 exon6F	CAACTTCAATCCCCAGTCCAAC	qPCR	WDR5 mRNA
WDR5 exon7R	TTGAGGCACTTCCCTGTTTTC	qPCR	WDR5 mRNA
RACE Primer AS-784	GGAGAATTTACCTTTCCCGCCTCTCT	RACE	COX-2 Promoter RNAs
RACE Primer AS-740	GGGTGAAGGTACGGAGAACAGTATTTC	RACE	COX-2 Promoter RNAs
RACE Primer AS-535	AACCTTACTCGCCCCAGTCTGTCC	RACE	COX-2 Promoter RNAs
RACE Primer AS-223	GGACTACCCCCTCTGCTCCCAAAT	RACE	COX-2 Promoter RNAs
RACE Primer AS-194	CAGCTTCCTGGGTTTCCGATTTTC	RACE	COX-2 Promoter RNAs
RACE Primer AS-65	GTCATTTCGTCACATGGGCTTGGT	RACE	COX-2 Promoter RNAs
RACE Primer AS-58	CGTCACATGGGCTTGGTTTTCAGT	RACE	COX-2 Promoter RNAs
RACE Primer AS-6	GCGACCAATTGTCATACGACTTGC	RACE	COX-2 Promoter RNAs
RACE Primer AS+9	TACGACTTGCAGTGAGCGTCAGGA	RACE	COX-2 Promoter RNAs
RACE Primer S+19	TGCAAGTCGTATGACAATTGGTCGCT	RACE	COX-2 Promoter RNAs
RACE Primer S+34	GCTCCTGACGCTCACTGCAAGT	RACE	COX-2 Promoter RNAs
RACE Primer S+98	TTTGCTGTCTGAGGGCGTCTGG	RACE	COX-2 Promoter RNAs
RACE Primer S+109	CGGGGGTAGGCTTTGCTGTCTGA	RACE	COX-2 Promoter RNAs
C1(-415F)	TCAGATTCCTGGAGAGGAAG	3C	COX-2 Promoter
C2(-946R)	TCCCTGATGCGTGGATTATTT	3C	COX-2 Promoter
C3(-1039R)	TGTCCACTTTTCCAAGATTATGAG	3C	COX-2 Promoter
T1(-139122F)	ACAGTGCCTACTATATGAAACAAAC	3C	Intergenic Region
T2(-144150R)	GTATTGCTTCCGTTGTTTAGGTC	3C	Intergenic Region
T3(-147737R)	GTAAGAGAGTTAGGAGAGTGAGTTC	3C	Intergenic Region
T4(-147867F)	CCCAAATTCCATGAGAATCAATTC	3C	PLA2G4A Promoter
T5(-148523R)	CCTACTCAGGATAAGACTTTCTC	3C	PLA2G4A Promoter
T6(148611F)	TGAGAATCTTCAGGCTCCTC	3C	PLA2G4A Promoter
T7(-154803R)	GTAAACTGGGATGTTCACCC	3C	PLA2G4A gene locus
T8(-158504F)	GAAAGATTTATGTCAGGGTGAAGAA	3C	PLA2G4A gene locus
T9	CGTCCTGTTTCAGAGTTTCCA	3C	PLA2G2E Promoter
T10	CACTCACGGGTTAATGACAGG	3C	PLA2G2E Promoter
T11	AGAATACCATTTACCTCCAGTGC	3C	PLA2G2A Promoter
T12	AATTCTTAGCATGTGCTGGAG	3C	PLA2G2A Promoter
Probe	6-FAM/ACCAGTATC/ZEN/TCCTATGA AGGGCTAGTAACC/IABkFQ	3C	COX-2 Promoter

Supplementary Table S2. Duplex RNAs used in this research.

Name	Sequence	Target (relative to +1 TSS for COX-2)
RNA9	UUAGCGACCAAUUGUCAUAdTdT	COX-2 (-9/+10)
RNA10	GUUAGCGACCAAUUGUCAUdTdT	COX-2 (-10/+9)
RNA11	GGUUAGCGACCAAUUGUCAdTdT	COX-2 (-11/+8)
RNA12	CGGUUAGCGACCAAUUGUCdTdT	COX-2 (-12/+7)
RNA13	UCGGUUAGCGACCAAUUGUdTdT	COX-2 (-13/+6)
RNA14	CUCGGUUAGCGACCAAUUGdTdT	COX-2 (-14/+5)
RNA34	CUUAUAAAAAGGAAGGUUCdTdT	COX-2 (-34/-16)
RNA38	CAGUCUUAUAAAAAGGAAGdTdT	COX-2 (-38/-20)
MM	UCGAAGUAUUCCGCGUACGdTdT	–
M1	CGGUUAGCGACGAUUAGACdTdT	–
M2	CGGUUAGCGACCAAUUCUCdTdT	–
M3	CCGUUACCGACGAAUUCUCdTdT	–
RNA12nc	CGGUUAGCGUGCAAUUGUCdTdT	COX-2 (-12/+7)
RNA12scr	UUGUCACCAACGGUUAGCGdTdT	–
RNA12m (sense)	GGGUuAGCGACcAAUUGUCdTdT	COX-2 (-12/+7)
RNA12m (antisense)	GAcAAUUGGUCGCuAACCGdTdT	COX-2 (-12/+7)
WDR5si	GUCGUCAGAUUCUAACCUUdTdT	WDR5 mRNA
AGO2si-1 (sense)	GCACGGAAGUCCAUCUGAAUU	AGO2 mRNA
AGO2si-1 (antisense)	pUUCAGAUGGACUUCCGUGCUU	AGO2 mRNA
AGO2si-2 (sense)	GCAGGACAAAGAUGUAUUAUU	AGO2 mRNA
AGO2si-2 (antisense)	pUAAUACAUCUUUGUCCUGCUU	AGO2 mRNA
AGO2si-3 (sense)	GGGUCUGUGGUGAUAAAUAUU	AGO2 mRNA
AGO2si-3 (antisense)	pUAUUUAUCACCACAGACCCUU	AGO2 mRNA
AGO2si-4 (sense)	GUAUGAGAACCCAAUGUCAUU	AGO2 mRNA
AGO2si-4 (antisense)	pUGACAUUGGGUUCUCAUACUU	AGO2 mRNA
TNRC6Asi (sense)	GCCAGAUGCCUAACAAUCA	TNRC6A (GW182) mRNA
TNRC6Asi (antisense)	UGAUUGUUAGGCAUCUGGCdTdT	TNRC6A (GW182) mRNA
TNRC6Bsi (sense)	GGACAAGCGAGCGAUGAAU	TNRC6B mRNA
TNRC6Bsi (antisense)	AUUCAUCGCUCGCUUGUCCdTdT	TNRC6B mRNA
TNRC6Csi (sense)	GAACCACAAACGUCCACUU	TNRC6C mRNA
TNRC6Csi (antisense)	AAGUGGACGUUUGUGGUUCdTdT	TNRC6C mRNA
miR-589 (active strand)	UGAGAACCACGUCUGCUCUGAG	COX-2 Promoter RNAs
miR-589 (carrier strand)	UCAGAACAAAUGCCGGUUCCCAGA	COX-2 Promoter RNAs
miR-589 mimic (active strand)	UGAGAACCACGUCUGCUCUGAG	COX-2 Promoter RNAs
miR-589 mimic (carrier strand)	CUCAGAGCAGACGUGGUUCUCA	COX-2 Promoter RNAs

RNA sequences are listed 5’ to 3’. Unless otherwise noted, only one (sense) strand of the duplex RNA is shown on this list. Mismatch bases are underlined. Small letters are 2’-O-methyl RNA nucleotides. Numbering of target site locations is relative to the +1 transcription start site for COX-2 mRNA. Each oligomer has a 2-nt deoxythymidine (dT) overhang at the 3’ end except for TNRC6Asi-6Csi sense strands and AGO2si. AGO2si-1, -2, -3, and -4 were used as an equimolar mixture. TNRC6Asi, 6Bsi, and 6Csi were also used as an equimolar mixture.

Supplementary Table S3. Single-stranded oligonucleotide gapmers.

Name	Sequence	Target (relative to +1 TSS for COX-2)
gap 1 (COX-2 G-48as)	CCCAUgtgacgaaatGACUG	Sense ncRNAs (-67/-48)
gap 2 (COX-2 G-67s)	CAGUCatttcgtcacAUGGG	Antisense ncRNAs (-67/-48)
Gapmer Control	CUUCUCagttgaattUAUAC	—

Uppercase letters are 2’-methoxyethyl RNA nucleotides. Lowercase letters are DNA.
